# Genome‐wide association study for 13 agronomic traits reveals distribution of superior alleles in bread wheat from the Yellow and Huai Valley of China

**DOI:** 10.1111/pbi.12690

**Published:** 2017-03-02

**Authors:** Congwei Sun, Fuyan Zhang, Xuefang Yan, Xiangfen Zhang, Zhongdong Dong, Dangqun Cui, Feng Chen

**Affiliations:** ^1^ Agronomy College/National Key Laboratory of Wheat and Maize Crop Science/Collaborative Innovation Center of Henan Grain Crops Henan Agricultural University Zhengzhou China; ^2^ Henan Key Laboratory of Nuclear Agricultural Sciences/Isotope Institute Co., Ltd Henan Academy of Sciences Zhengzhou China

**Keywords:** Bread wheat (*Triticum aestivum* L.), GWAS, QTL mapping, Wheat 90K SNP assay, Yield‐related traits, Superior allele

## Abstract

Bread wheat is a leading cereal crop worldwide. Limited amount of superior allele loci restricted the progress of molecular improvement in wheat breeding. Here, we revealed new allelic variation distribution for 13 yield‐related traits in series of genome‐wide association studies (GWAS) using the wheat 90K genotyping assay, characterized in 163 bread wheat cultivars. Agronomic traits were investigated in 14 environments at three locations over 3 years. After filtering SNP data sets, GWAS using 20 689 high‐quality SNPs associated 1769 significant loci that explained, on average, ~20% of the phenotypic variation, both detected already reported loci and new promising genomic regions. Of these, repetitive and pleiotropic SNPs on chromosomes 6AS, 6AL, 6BS, 5BL and 7AS were significantly linked to thousand kernel weight, for example BS00021705_51 on 6BS and wsnp_Ex_c32624_41252144 on 6AS, with phenotypic variation explained (PVE) of ~24%, consistently identified in 12 and 13 of the 14 environments, respectively. Kernel length‐related SNPs were mainly identified on chromosomes 7BS, 6AS, 5AL and 5BL. Plant height‐related SNPs on chromosomes 4DS, 6DL, 2DS and 1BL were, respectively, identified in more than 11 environments, with averaged PVE of ~55%. Four SNPs were confirmed to be important genetic loci in two RIL populations. Based on repetivity and PVE, a total of 41 SNP loci possibly played the key role in modulating yield‐related traits of the cultivars surveyed. Distribution of superior alleles at the 41 SNP loci indicated that superior alleles were getting popular with time and modern cultivars had integrated many superior alleles, especially for peduncle length‐ and plant height‐related superior alleles. However, there were still 19 SNP loci showing less than percentages of 50% in modern cultivars, suggesting they should be paid more attention to improve yield‐related traits of cultivars in the Yellow and Huai wheat region. This study could provide useful information for dissection of yield‐related traits and valuable genetic loci for marker‐assisted selection in Chinese wheat breeding programme.

## Introduction

Bread wheat (*Triticum aestivum* L.) is one of the most important crops worldwide. Given the gradual decrease in farmland and rapid increase in global population, high yield is always one of the most important targets in wheat breeding. Wheat yield consists of three key components: thousand kernel weight (TKW), spike number/m^2^ and kernel number per spike (KNS). Moreover, other agronomic traits also play important roles in determination of wheat yield, for example plant height (PH), spike length (SL), kernel weight per spike (KWS), kernel length (KL) and kernel width (KW). Larger kernels are not only directly related to wheat yield but also have favourable effects on seeding vigour and early growth (Peng *et al*., 2003). The semi‐dwarf cultivars improved by *Rht1* and *Rht2* were major contributors to the success of the Green Revolution (Ellis *et al*., 2005). To date, many QTLs associated with yield‐related traits in bread wheat have been identified by biparental QTL mapping (Gao *et al*., 2015; Milner *et al*., 2016; Yu *et al*., 2016) and a number of genes been isolated by *in silico* cloning in bread wheat (Hou *et al*., 2014; Li *et al*., 2016; Ma *et al*., 2015; Wang *et al*., 2015, 2016; Zheng *et al*., 2014). For example, *TaGS5* gene was isolated in bread wheat and was found to regulate grain size and weight (Ma *et al*., 2015; Wang *et al*., 2015, 2016). Two wheat sucrose synthase genes (*TaSus1* and *TaSus2* on chromosomes 7 and 2, respectively) were revealed to be associated with TKW in Chinese cultivars in multiple environments (Hou *et al*., 2014). Yu *et al*. (2016) constructed an integrated genetic map containing 926 molecular markers for einkorn wheat A genome and provided valuable information for QTL mapping and genome sequence anchoring. However, the standard QTL mapping methods only locate associated genomic regions with low resolution, and not take diverse genetic background into consideration (Sukumaran *et al*., 2015).

As a complement to QTL mapping, GWAS, based on linkage disequilibrium (LD), provide the opportunity to methodically analyse the genetic architecture of complex traits in crop plants (Nordborg and Weigel, 2008). Up to now, great progress on GWAS has been achieved in rice (Huang *et al*., 2010), maize (Li *et al*., 2013), foxtail millet (Jia *et al*., 2013) and sesame (Wei *et al*., 2015). Compared with the genome of rice (≈400 Mb) and maize (≈3 Gb), hexaploid wheat has a larger genome size (≈17.9 Gb; Varshney *et al*., 2006). Absence of a completely sequenced reference genome has limited gene discovery in bread wheat in the last decade (Pingault *et al*., 2015). In previous studies on polyploidy wheat, the association panels were usually characterized by simple sequence repeat (SSR) markers and limited SNP numbers (Lopes *et al*., 2014). Maccaferri *et al*. (2011) dissected the genetic basis of drought‐adaptive traits and grain yield in durum wheat accessions by association mapping and found that Xbarc197 on chromosome 5A was significantly associated with grain yield in six environments. A diverse elite European winter wheat panel was assessed with both the 9K iSelect Beadchip Assay and 91 SSR markers, and the results suggested that SNPs were more valuable for knowledge‐based improvement of wheat and that more SNPs were needed to obtain a higher coverage of the wheat genome (Wurschum *et al*., 2013).

The newly developed wheat 90K SNP genotyping assay comprised approximately 90 000 gene‐associated SNPs covering the whole genome. Compared to SSR marker and the 9K iSelect Beadchip Assay, the 90K SNP assay is generally more abundant, stable, amenable to automation, efficient and cost‐effective (Wang *et al*., 2014). Recent findings based on the 90K SNP genotyping assay can be found in biparental QTL mapping (Avni *et al*., 2014; Gao *et al*., 2015; Liu *et al*., 2015) and GWAS for yield‐related traits in spring wheat population grown in temperate irrigated environments (Sukumaran *et al*., 2015), under rainfed conditions in Pakistan historical wheat cultivars (Ain *et al*., 2015), in European wheat cultivars (Guo *et al*., 2016; Zanke *et al*., 2015), and in Ethiopian durum wheat landraces (Mengistu *et al*., 2016).

China is the largest wheat producer after the European Union. Chinese wheat is mainly planted in ten agro‐ecological zones, in which the Yellow and Huai wheat region is the most important and largest wheat production zone with 60%–70% of both total harvested area and total wheat production (Chen *et al*., 2013). Cultivars of this region have made great contribution to the national wheat yield, and the traditional breeding method used in this region has experienced limited improvement in last decade. Therefore, we selected 163 representative bread wheat cultivars with relatively distant genetic relationship, and made series of GWAS for 13 important agronomic traits in 14 environments within three locations over 3 years. The aim of GWAS is to reveal new allelic variation affecting wheat yield and find their superior alleles for breeders to molecular improvement in the Yellow and Huai Valley of China.

## Results

### Population structure and linkage disequilibrium analysis

Before GWAS, the 90K SNP genotyping assay was filtered in PLINK software, about 36 916 SNPs failed frequency test (MAF < 0.02), 4104 SNPs failed missing test (GENO > 0.1), and 2990 SNPs were excluded based on HWE test (*P *≤ 1.0e−9). After frequency and genotyping pruning, there are 20 689 SNPs. No individual was removed for low genotyping (MIND > 0.1). Finally, 163 cultivars with 20 689 SNPs were retained for further analysis.

**Figure 1:**
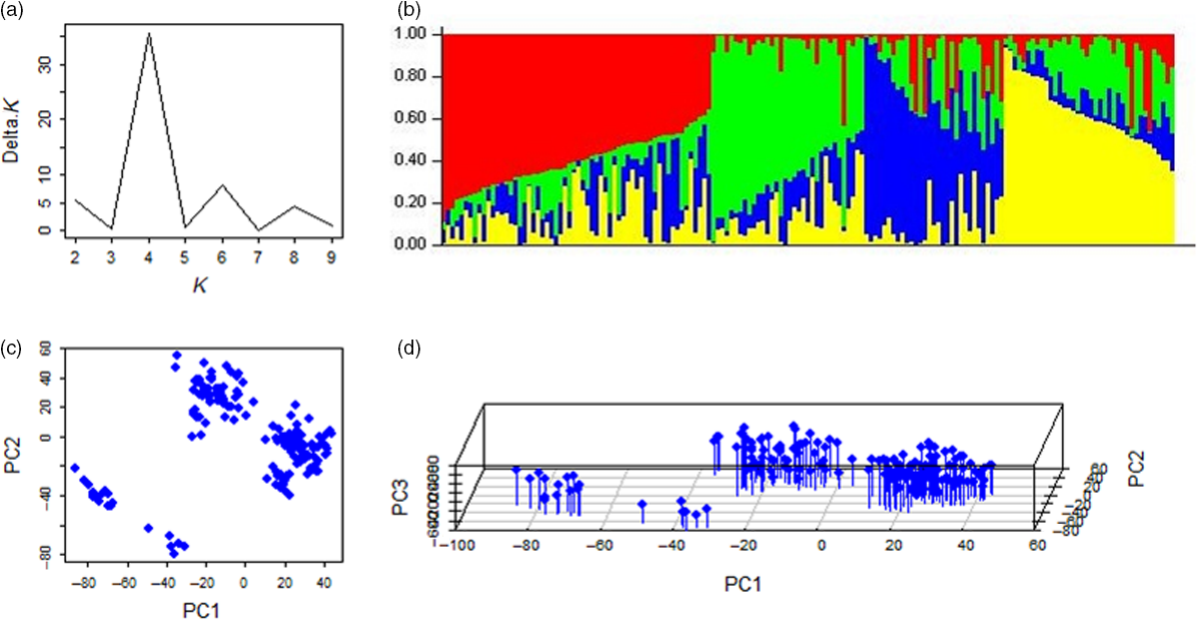
Population structure of the natural population based on unlinked SNP markers. (a) Plot of delta K against putative K ranging from 1 to 10; (b) stacked bar plot of ancestry relationship of 163 cultivars; (c) plot of first principle component against second principle component; (d) plot of first principle component against second and third principle components.

The number of subpopulation (*k*) was plotted against the delta *k* calculated from the STRUCTURE software (Figure 1a). The peak of the broken line graph was observed at *k* = 4, indicating that the natural population can be divided into four subpopulations (Figure 1b). The PCA also classified the population into four subpopulations, which supported the inference of STRUCTURRE software (Figure 1c, d).

Linkage disequilibrium was calculated for A, B and D genome, using 7016, 8240 and 5433 SNP markers, respectively. Highest linkage disequilibrium was found in the A genome, followed by D and B genomes. Linkage disequilibrium delayed below *r* ^2^ = 0.02 at about 231 607 base pair in A genome, 173 750 base pair in D genome and 132 889 base pair in B genome.

### Significant and repetitive loci associated with agronomic traits

**Figure 2:**
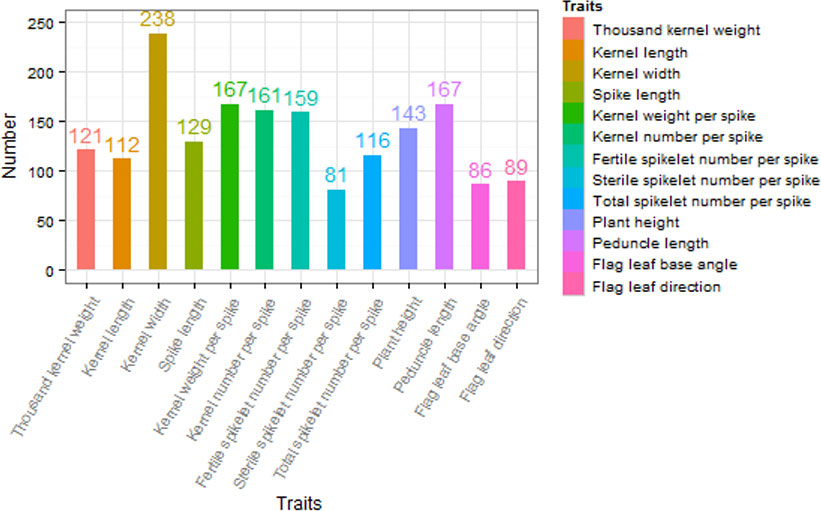
The numbers of associated significant SNPs for 13 agronomic traits.

The investigated traits in the natural population showed abundant phenotypic variation in the surveyed environments (Figure S1). Association studies were performed with kernel‐related traits, panicle‐related traits and plant architecture‐related traits and totally identified 1769 significant SNPs at a suggestive *P *< 1.0e−3 for 13 agronomic traits in 14 environments. The number of associated loci for each trait varied (Figure 2), which on average explained ~20% of the phenotypic variation. Based on significance and repetivity of SNPs associated with 13 agronomic traits in 14 environments, some important SNPs are summarized in Table 1, and their comparison with previous studies is shown in Table 2.

**Table 1:** Repetitive SNPs and their *P* values revealed by GWAS consistently identified in more than half of the investigated environments

Traits	SNP name	Chr	Position	2012–2013						2013–2014						2014–2015	
				Anyang		Zhengzhou		Zhumadian		Anyang		Zhengzhou		Zhumadian		Zhengzhou	
				E1	E2	E3	E4	E5	E6	E7	E8	E9	E10	E11	E12	E13	E14
TKW	wsnp_Ex_c32624_41252144	6AS	4382409	3.7E−04	1.1E−04	2.3E−04	8.0E−04	2.8E−04	4.1E−04	4.3E−04	2.1E−04	1.8E−04	1.5E−05	1.2E−04	1.3E−04	1.8E−04	ns
	BS00021705_51	6BS	3017544	3.2E−04	8.0E−05	3.4E−04	1.5E−04	ns	2.0E−04	2.0E−05	6.8E−04	5.1E−04	2.4E−04	5.9E−04	4.8E−04	8.8E−05	ns
KL	BS00036788_51	7BS	3144105	2.4E−04	4.5E−04	5.7E−04	5.2E−04	ns	ns	ns	3.2E−05	5.6E−04	6.1E−04	4.6E−05	6.3E−06	9.5E−05	ns
	BS00010573_51	5AL	2796335	ns	5.5E−04	4.6E−04	ns	4.3E−04	ns	4.6E−04	3.0E−04	1.4E−04	6.9E−04	1.8E−04	ns	ns	ns
	IACX9238	5BL	10878230	ns	7.5E−04	5.5E−04	ns	5.2E−04	ns	7.8E−04	4.4E−04	2.6E−04	8.9E−04	2.4E−04	ns	ns	ns
	Kukri_c2951_2574	6AS	4394696	1.6E−04	3.1E−04	ns	ns	ns	ns	ns	ns	4.6E−04	3.0E−04	4.2E−04	8.8E−04	7.5E−04	ns
	BS00003733_51	3AS	3443101	ns	ns	ns	ns	6.1E−04	ns	ns	4.5E−04	3.7E−04	5.1E−05	2.2E−04	2.7E−04	1.3E−04	ns
	BobWhite_c9961_402	3B	10681096	ns	ns	ns	ns	6.1E−04	ns	ns	4.5E−04	3.7E−04	5.1E−05	2.2E−04	2.7E−04	1.3E−04	ns
	BS00011516_51	3DS	2602042	ns	ns	ns	ns	6.1E−04	ns	ns	4.5E−04	3.7E−04	5.1E−05	2.2E−04	2.7E−04	1.3E−04	ns
KNS	Kukri_c4586_381	6BS	1011973	3.1E−04	ns	ns	ns	ns	6.5E−04	ns	5.2E−04	1.3E−04	4.0E−04	5.7E−04	1.7E−05	ns	6.6E−04
SL	BS00022060_51	2BS	5247056	4.4E−06	2.6E−04	2.1E−04	1.6E−05	4.1E−06	1.3E−05	2.7E−06	3.6E−05	5.2E−06	ns	5.8E−04	6.7E−05	8.1E−04	ns
	BS00085688_51	6BL	4397705	7.9E−06	3.3E−05	6.7E−07	4.2E−06	7.4E−06	2.9E−05	6.7E−05	1.5E−05	1.3E−05	ns	2.4E−05	2.7E−05	4.7E−05	9.6E−04
	BS00046263_51	6DL	3219530	1.1E−05	4.0E−05	7.1E−07	5.0E−06	1.5E−05	3.6E−05	7.4E−05	1.6E−05	9.1E−06	ns	2.6E−05	2.9E−05	8.1E−05	9.7E−04
	CAP12_rep_c3980_87	2BL	7941882	5.5E−05	1.2E−06	1.7E−05	2.8E−05	2.0E−05	1.5E−06	6.1E−05	9.9E−05	9.9E−06	8.1E−06	4.5E−05	1.4E−04	3.1E−06	ns
	Excalibur_c37787_925	3B	10739251	2.0E−04	5.0E−05	1.3E−05	2.6E−05	4.7E−04	ns	ns	4.7E−04	1.7E−04	ns	4.9E−04	1.8E−04	ns	ns
	BobWhite_rep_c66146_237	1BL	3892394	2.6E−04	1.4E−04	1.7E−04	ns	9.3E−04	2.8E−05	1.1E−04	5.7E−04	7.3E−04	ns	1.5E−04		ns	ns
PH	Kukri_rep_c68594_530	4DS	2311430	7.2E−04	4.0E−04	6.8E−05	1.2E−04	2.2E−04	1.6E−04	1.8E−04	6.3E−05	2.8E−04	4.4E−04	8.2E−05	8.6E−04	3.1E−04	ns
	Tdurum_contig29489_176	6DL	3262410	7.0E−05	5.9E−05	ns	8.0E−04	1.3E−05	9.7E−06	1.5E−05	2.1E−05	2.7E−05	1.1E−06	5.5E−04	1.8E−04	ns	ns
	Tdurum_contig27385_131	1BL	3915948	3.0E−04	4.5E−04	2.1E−04	2.9E−04	5.5E−04	2.2E−04	3.7E−04	3.2E−04	3.0E−04	9.5E−04	ns	4.2E−04	ns	ns
	RAC875_c48703_148	2DS	5358861	1.0E−04	3.0E−04	4.4E−05	2.1E−04	ns	ns	7.5E−05	2.1E−04	2.5E−06	5.2E−07	3.5E−05	1.4E−04	4.8E−04	ns
PL	Kukri_c5282_622	2AS	5204476	1.4E−04	ns	1.8E−04	1.2E−04	ns	5.0E−04	ns	ns	ns	2.0E−04	3.1E−04	1.6E−04	ns	ns
	RAC875_c829_611	2AS	5234765	1.4E−04	ns	1.8E−04	1.2E−04	ns	5.0E−04	ns	ns	ns	2.0E−04	3.1E−04	1.6E−04	ns	ns
	TA006231‐0789	7AL	4472650	1.2E−04	3.8E−04	ns	6.2E−04	ns	ns	5.6E−04	3.3E−04	ns	ns	ns	9.2E−04	ns	ns
FLBA	wsnp_Ex_rep_c67561_66189356	2BL	7961027	6.4E−04	ns	ns	1.8E−04	3.3E−04	3.2E−04	3.9E−04	6.9E−04	ns	4.8E−04	7.6E−04	6.3E−04	–	–
FLD	Tdurum_contig12831_69	2DL	9888404	ns	ns	ns	ns	1.1E−04	9.4E−04	6.4E−04	6.4E−04	5.6E−04	5.6E−04	4.9E−04	8.7E−04	–	–
	wsnp_Ex_rep_c67561_66189356	2BL	7961027	ns	ns	ns	ns	1.7E−04	ns	5.7E−04	5.7E−04	1.8E−04	1.8E−04	1.7E−04	3.3E−04	–	–

TKW, thousand kernel weight; KL, kernel length; KNS, kernel number per spike; SL, spike length; PH, plant height; PL, peduncle length; FLBA, flag leaf base angle; FLD, flag leaf direction.

‘–’, indicated that data were missing.

ns, indicate not significant.

**Table 2:** Significant and repetitive SNP loci identified in current and previous study

Trait	Chromosome	Identified loci in current study	Near locus previously reported in the same chromosome
TKW	3A	Excalibur_c39508_88	*TaGS5* (Ma *et al*., 2015; Wang *et al*., 2015, 2016)
	5B	Jagger_c4951_122	wsnp_Ex_rep_c66651_64962429~TA002629‐0202 (Ain *et al*., 2015)
	5B	Excalibur_c23801_115	wpt3457 (Edae *et al*., 2014)
	5B	BS00060460_51	
	6A	wsnp_Ex_c32624_41252144	*TaGW2* (Bednarek *et al*., 2012)
	6A	Ra_c90_3168	Ku_c6998_485 (Gao *et al*., 2015)
	6A	Tdurum_contig61574_645	RFL_Contig4632_1512 (Sukumaran *et al*., 2015)
	6A	Tdurum_contig55363_297	(Lopes *et al*., 2014)
	6A	Tdurum_contig29974_90	
	6A	Ku_c6998_485	
	6B	BS00021705_51	
	7A	IAAV416	IWB319–IWB23989 (Milner *et al*., 2016)
KL	3A	BS00003733_51	wPt1688‐barc45 (Gegas *et al*., 2010)
	3A		wPt9562‐wPt9215 (Gegas *et al*., 2010)
	3A		barc19‐cfa2262 (Gegas *et al*., 2010)
	3B	BobWhite_c9961_402	
	3D	BS00011516_51	*TaCKX2* (Zhang *et al*., 2011)
	5A	BS00010573_51	gwm443‐cfa2104 (Gegas *et al*., 2010)
	5A		wmc492‐wmc475 (Gegas *et al*., 2010)
	5A		wPt0654‐wPt3069 (Gegas *et al*., 2010)
	5A		wmc110‐wmc727 (Gegas *et al*., 2010)
	5B	IACX9238	*Vrn‐B1* (Yan *et al*., 2003)
	6A	Kukri_c2951_2574	wmc6807‐psp3071 (Gegas *et al*., 2010)
	6A		cos07 Tb‐wPt5480 (Gegas *et al*., 2010)
	7B	BS00036788_51	
KNS	2D	Ra_c72517_981	Kukri_c14902_1112‐RAC875_c77816_365 (Gao *et al*., 2015)
	2D		kukri_rep_c106786_230 (Guo *et al*., 2016)
	6B	Kukri_c4586_381	RAC875_c28848_330‐BS00065202_51 (Gao *et al*., 2015)
SL	1B	BobWhite_rep_c66146_237	IAAV4702‐wsnp_BG274294B_Ta_2_3 (Gao *et al*., 2015)
	2B	CAP12_rep_c3980_87	
	2B	BS00022060_51	
	3B	Excalibur_c37787_925	Ku_c12191_1202‐Excalibur_c3556_520 (Gao *et al*., 2015)
	6B	BS00085688_51	Ra_c2557_2531‐BS00067417_51 (Gao *et al*., 2015)
	6D	BS00046263_51	
PH	1B	Tdurum_contig27385_131	IWB66840–IWB8804 (Milner *et al*., 2016)
	2D	RAC875_c48703_148	*Ppd‐D1* (Guo *et al*., 2010)
	4D	Kukri_rep_c68594_530	Kukri_rep_c68594_530 (Chen *et al*., 2015)
	4D		RAC875_c13945_597‐BS00036421_51 (Gao *et al*., 2015)
	4D		
	4B	Tdurum_contig29563_183	RAC875_c86104_111‐tplb0025f09_1853 (Gao *et al*., 2015)
	4B		RAC875_c6749_954‐BobWhite_c44691_648 (Gao *et al*., 2015)
	6D	Tdurum_contig29489_176	
PL	2A	Kukri_c5282_622	
	2A	RAC875_c829_611	
	7A	TA006231‐0789	
FLBA	2B	wsnp_BF473744B_Ta_2_2	
	2B	wsnp_Ex_rep_c67561_66189356	
	4D	Excalibur_c19547_1012	
	5A	Ra_c71978_532	
FLD	2B	GENE‐0675_104	
	2D	Tdurum_contig12831_69	

TKW, thousand kernel weight; KL, kernel length; KNS, kernel number per spike; SL, spike length; PH, plant height; PL, peduncle length; FLBA, flag leaf base angle; FLD, flag leaf direction.

### Kernel‐related traits

#### Thousand kernel weight

**Figure 3:**
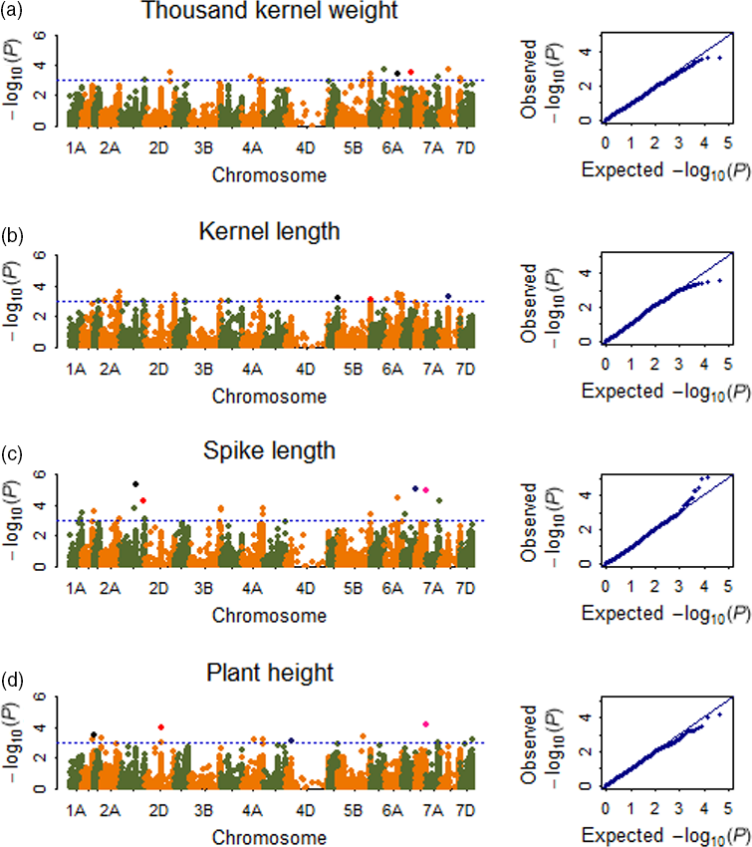
Manhattan and Q‐Q plots for TKW, kernel length, spike length and PH. (a) Thousand kernel weight (TKW), the black and red dots represent SNP named wsnp_Ex_c32624_41252144 and BS00021705_51, consistently detected in 13 and 12 environments, respectively; (b) kernel length (KL), the black, red and blue dots represent SNP named BS00036788_51, BS00010573_51 and IACX9238, consistently detected in ten, eight and eight environments, respectively; (c) spike length (SL), the black, red, blue and pink dots represent SNP named BS00085688_51, BS00046263_51, CAP12_rep_c3980_87 and BS00022060_51, consistently detected in 13, 13, 13 and 12 environments, respectively; (d) plant height (PH), the black, red, blue and pink dots represent SNP named Kukri_rep_c68594_530, Tdurum_contig29489_176, RAC875_c48703_148 and Tdurum_contig27385_131, consistently detected in 13, 11, 11 and 11 environments, respectively.

A total of 121 significant SNPs, mainly distributed on chromosomes 6AS, 6AL, 6BS, 7AL, 7AS, 5BL, 5DL, 3AL, 3DL and 1AL, were associated with TKW (Tables 1, S2; Figures 3a, S2). The phenotypic variation explained (PVE) by each SNP ranged from 15.7% to 31.6%. Of all significant SNPs, wsnp_Ex_c32624_41252144 on 6AS and BS00021705_51 on 6BS were detected to be significantly associated with TKW in 13 and 12 environments with averaged PVE of 24.6% and 24.1%, respectively. The wsnp_Ex_c32624_41252144 showed a positive effect on TKW, while the BS00021705_51 showed a negative effect on TKW. Besides, Tdurum_contig61574_645, Tdurum_contig55363_297 and Tdurum_contig29974_90 on chromosome 6AL were detected in the same five environments, with averaged PVE of 23.8%. Of these, Tdurum_contig61574_645 showed a positive effect on TKW, and Tdurum_contig55363_297 and Tdurum_contig29974_90 showed negative effect on TKW.

#### Kernel size

A total of 112 significant SNPs, mainly distributed on chromosomes 7BS, 7BL, 6AS, 6AL, 5AL, 5BL, 4AS, 3AS, 3B, 3DS 2BL and 2AL, were associated with kernel length (Tables 1, S2; Figures 3b, S2). The phenotypic variation explained by each SNP ranged from 20.9% to 42.2%. Of all significant SNPs, BS00036788_51 on 7BS (with PVE of 29.0%), BS00010573_51 on 5AL (with PVE of 26.7%), IACX9238 on 5BL (with PVE of 26.4%), Kukri_c2951_2574 on 6AS (with PVE of 29.0%), BS00003733_51 on 3AS (with PVE of 29.3%), BobWhite_c9961_402 on 3B (with PVE of 29.3%) and BS00011516_51 on 3DS (with PVE of 29.3%) were detected to be significantly associated with kernel length in ten, eight, eight, seven, six, six and six environments, respectively. Kukri_c2951_2574, BS00003733_51 and BobWhite_c9961_402 showed positive effect on kernel length, while BS00036788_51, BS00010573_51, IACX9238 and BS00011516_51 showed negative effect on kernel length. Identification of *Vrn‐B1* gene in the natural population indicated that cultivars with dominant allele *Vrn‐B1* showed significantly longer kernel length than those with recessive allele *vrn‐B1* in all the investigated environments.

A total of 238 significant SNPs, mainly distributed on chromosomes 6AS, 6AL, 6BS, 5AS, 4AS, 4DS, 4BS and 2DL, were associated with kernel width (Table S2; Figure S2). The number of significant SNPs detected in kernel width was larger than any other traits and had poor repeatability. The phenotypic variation explained by each SNP ranged from 9.4% to 32.1%. To decrease manual errors in measuring kernel width, SKCS was used to remeasure kernel diameter. Significant SNPs associated with kernel diameter were mainly observed on chromosomes 6AL, 6BL, 5AS, 5DL, 4AL, 4AS and 1BL, of these, Kukri_c85856_60 on 6BL, wsnp_Ex_c4436_7981188 on 1BL and BS00047195_51 on 5AS, with averaged PVE of 28.6%, 26.0% and 19.8%, respectively, in three of the seven environments (seeds from the same location and the same year were mixed together for SKCS).

### Panicle‐related traits

#### Spike length

A total of 129 significant SNPs, mainly distributed on chromosomes 6AS, 6BL, 6DL, 3B, 2BL, 2BS and 1BL, were associated with spike length (Tables 1, S2; Figures 3c, S2). The phenotypic variation explained by each SNP ranged from 4.4% to 21.0%. Of all significant repetitive SNPs, BobWhite_rep_c66146_237 on 1BL (with PVE of 10.2%) detected in nine environments showed a positive effect on spike length, and the remaining five SNPs in 9–13 environments showed negative effect on spike length.

#### Kernel weight per spike

A total of 167 significant SNPs, mainly distributed on chromosomes 7DL, 7DS, 6AS, 6AL, 6BS, 6DL, 5BL and 3B, were associated with KWS (Table S2; Figure S2). The phenotypic variation explained by each SNP ranged from 13.0% to 22.6%. Repetitive SNPs BS00096853_51 showing a negative effect (with PVE of 29.3%), Ku_c47648_1403 (with PVE of 29.3%) and GENE‐3703_114 showing a positive effect (with PVE of 29.3%) were detected in six, five and five environments, respectively.

#### Kernel number per spike

A total of 161 significant SNPs, mainly distributed on chromosomes 7BL, 7DL, 6BS, 4DS, 3AL, 3DS, 2AL, 2BL and 2DL, were associated with KNS (Tables 1, S2; Figure S2). The phenotypic variation explained by each SNP ranged from 5.4% to 20.2%. Of all significant SNPs, Kukri_c4586_381 on 6BS was detected to be significantly associated with KNS in eight environments with averaged PVE of 11.7%, and showed a negative effect on KNS.

#### Spikelet number per spike

A total of 116 significant SNPs, mainly distributed on chromosomes 7AL, 6AS, 6BS, 3AL, 3AS, 3DL, 3DS, 2BS, 1AL and 1BL, were associated with TSNS (Table S2; Figure S2). The phenotypic variation explained by each SNP ranged from 15.5% to 33.7%. Repetitive SNP Kukri_c264_539, Tdurum_contig42729_380 and tplb0059e14_515 on 6AS, 6AS and 1AL were detected in four environments with an averaged PVE of 28.3%, 23.1% and 23.5%, respectively.

In addition, a total of 159 significant SNPs, mainly distributed on chromosomes 6AS, 6BS, 3AL, 3DL, 3DS, 2BL and 1BL, were associated with FSNS (Table S2; Figure S2), and their phenotypic variation explained by each SNP ranged from 10.3% to 27.0%. A total of 81 significant SNPs, mainly distributed on chromosomes 7AL, 6BS, 6DS, 5BL, 4BL, 3DL, 2BS, 2AL, 1BL, 1BS, 1DL and 1DS, were associated with SSNS (Table S2; Figure S2), and their phenotypic variation explained by each SNP ranged from 7.9% to 29.7%.

### Plant architecture‐related traits

#### Plant height

A total of 143 significant SNPs, mainly distributed on chromosomes 6DL, 4DS, 4BS, 2AS, 2BS, 2DS and 1BL, were associated with plant height (Tables 1, S2; Figures 3d, S2). The phenotypic variation explained by each SNP ranged from 35.8% to 63.6%. Of all significant SNPs, Kukri_rep_c68594_530 on 4DS (with PVE of 54.4%), Tdurum_contig29489_176 on 6DL (with PVE of 55.8%), Tdurum_contig27385_131 on 1BL (with PVE of 55.4%), RAC875_c48703_148 on 2DS (with PVE of 54.3%), Tdurum_contig29563_183 on 4BS (with PVE of 50.4%), RAC875_c48625_311 on 2BS (with PVE of 52.1%) and BS00010629_51 on 2AS (with PVE of 51.5%) were detected to be significantly associated with plant height in 13, 11, 11, 11, seven, six and six environments, respectively. Identification of *Rht‐D1* gene in the natural population and the two RIL populations indicated that cultivars with *Rht‐D1b* showed significantly lower plant height than those with *Rht‐D1a*, possibly indicating that Kukri_rep_c68594_530 was closely linked to *Rht‐D1* gene due to their same chromosome location. Identification of *Ppd‐D1* gene in the natural population showed that *Ppd‐D1* gene was also significantly associated with plant height in all the investigated environments, and cultivars with *Ppd‐D1b* showed significantly higher plant height than cultivars with *Ppd‐D1a*.

#### Peduncle length

A total of 167 significant SNPs, mainly distributed on chromosomes 7AL, 7DS, 4BS, 3AL, 2DS, 2AS, 2AL and 1BL, were associated with peduncle length (Tables 1, S2; Figure S2). The phenotypic variation explained by each SNP ranged from 26.5% to 49.9%. Of all significant SNPs, Kukri_c5282_622 (with PVE of 38.7% and positive effect) and RAC875_c829_611 (with PVE of 38.7% and negative effect) both on 2AS, and TA006231‐0789 on 7AL (with PVE of 41.2% and positive effect) were detected to be significantly associated with peduncle length in eight, eight and six environments.

#### Flag leaf base angle

A total of 86 significant SNPs, mainly distributed on chromosomes 5AL, 5BL, 2AL 2BL and 2DL, were associated with FLBA (Tables 1, S2; Figure S2). The phenotypic variation explained by each SNP ranged from 23.7% to 33.9%. Of all significant SNPs, wsnp_Ex_rep_c67561_66189356 on 2BL and Ra_c71978_532 on 5AL both explained 29.2% of the phenotypic variation and detected in nine environments. Ra_c71978_532 showed a positive effect on FLBA, while wsnp_Ex_rep_c67561_66189356 showed a negative effect on FLBA.

#### Flag leaf direction

A total of 89 significant SNPs, mainly observed on chromosomes 6BS, 5AL, 3B, 2AL, 2BL and 2DL, were associated with FLD (Tables 1, S2; Figure S2). The phenotypic variation explained by each SNP ranged from 19.9% to 31.5%. Of these, Tdurum_contig12831_69 on 2DL (with PVE of 24.4%) was significantly positively associated with FLD in eight environments, and wsnp_Ex_rep_c67561_66189356 on 2BL (with PVE of 25.1%) was significantly negatively associated with FLD in seven environments. BS00040143_51 on 3B explained the highest phenotypic variation and showed a positive effect on FLD.

### Validation of candidate SNPs in natural population

We verified the candidate repetitive SNPs in the natural population based on their polymorphism (Table 3), and the differences of phenotypic values grouped by polymorphism reached extremely significant levels (*P *< 0.01).

**Table 3:** *P* values of *t*‐test for repetitive candidate SNPs in different traits

Traits	SNPname	Chr	Allele		Number		Phenotype		2013						2014						2015	
									Anyang		Zhengzhou		Zhumadian		Anyang		Zhengzhou		Zhumadian		Zhengzhou	
									E1	E2	E3	E4	E5	E6	E7	E8	E9	E10	E11	E12	E13	E14
TKW	BS00021705_51	6BS	AC	CC	81	75	50.2	47.1	0.000	0.000	0.001	0.002	0.001	0.001	0.000	0.001	0.000	0.000	0.001	0.000	0.000	0.012
	Jagger_c4951_122	5BL	TC	TT	69	93	47.5	50.2	0.000	0.003	0.001	0.003	0.009	0.002	0.001	0.018	0.022	0.014	0.002	0.003	0.081	0.039
	Excalibur_c23801_115	5BL	TT	TC	124	39	48.1	51.9	0.002	0.002	0.000	0.000	0.001	0.000	0.001	0.015	0.001	0.002	0.001	0.003	0.017	0.071
KL	BS00010573_51	5AL	AC	CC	62	100	7.2	6.9	0.000	0.000	0.001	0.016	0.001	0.006	0.000	0.001	0.001	0.001	0.000	0.002	0.008	0.003
	IACX9238	5BL	AC	CC	63	100	7.2	6.9	0.001	0.000	0.001	0.016	0.001	0.007	0.001	0.001	0.001	0.001	0.000	0.002	0.008	0.002
	Kukri_c2951_2574	6AS	AG	GG	79	75	6.9	7.1	0.003	0.010	0.005	0.002	0.001	0.000	0.004	0.004	0.001	0.001	0.001	0.002	0.000	0.032
PH	Kukri_rep_c68594_530	4DS	AA	AG	70	91	87.4	78.3	0.000	0.000	0.000	0.000	0.000	0.000	0.000	0.000	0.000	0.000	0.000	0.000	0.000	0.007
SL	BS00085688_51	6BL	CC	TC	100	62	10.2	9.3	0.000	0.000	0.000	0.000	0.000	0.000	0.000	0.000	0.000	0.000	0.000	0.000	0.000	0.000
	BS00046263_51	6DL	CC	TC	100	58	10.2	9.3	0.000	0.000	0.000	0.000	0.000	0.000	0.000	0.000	0.000	0.000	0.000	0.000	0.000	0.000
	BobWhite_rep_c66146_237	1BL	TG	GG	51	110	10.3	9.7	0.002	0.002	0.004	0.004	0.005	0.000	0.000	0.001	0.003	0.041	0.010	0.119	0.033	0.881
PL	TA006231‐0789	7AL	CC	TC	97	64	26.0	29.1	0.000	0.000	0.000	0.000	0.000	0.000	0.000	0.000	0.000	0.000	0.000	0.000	0.000	0.000
FLBA	wsnp_Ex_rep_c67561_66189356	2BL	TT	TC	78	85	2.7	2.2	0.000	0.000	0.000	0.000	0.000	0.000	0.000	0.000	0.000	0.000	0.000	0.000	–	–
FLD	Tdurum_contig12831_69	2DL	AC	CC	79	83	1.7	1.4	0.006	0.001	0.001	0.001	0.000	0.000	0.000	0.000	0.000	0.000	0.000	0.000	–	–
	wsnp_Ex_rep_c67561_66189356	2BL	TT	TC	78	85	1.7	1.4	0.005	0.001	0.001	0.001	0.000	0.000	0.000	0.000	0.000	0.000	0.000	0.000	–	–

TKW, thousand kernel weight; KL, kernel length; SL, spike length; PH, plant height; PL, peduncle length; FLBA, flag leaf base angle; FLD, flag leaf direction.

‘–’, indicated that data were missing.

Among the significant SNPs, some of them were associated with more than one trait. We identified the significant SNPs for pairwise traits and summarized the numbers (Table S3). On the basis of pairwise traits, we identified pleiotropic SNPs associated with three or more traits. We also made a validation based on the polymorphism for pleiotropic SNPs (Table 4). As shown in Table 4, cultivars with allele AG in Excalibur_c39508_88 showed higher TKW, KWS and more FSNS, whereas cultivars with allele GG in Excalibur_c19547_1012 showed more SSNS and smaller FLBA. Cultivars with allele GG in BS00060460_51 showed higher TKW, KWS and longer kernel length. Cultivars with allele AG in Ra_c72517_981 showed higher KWS and more KNS. Cultivars with allele TT in Jagger_c4951_122 showed higher TKW and KWS and longer spike length.

**Table 4:** *P* values of *t*‐test for pleiotropic candidate SNPs in different traits

SNPname	Chr	Allele		Number		Phenotype		Traits	2013						2014						2015	
									Anyang		Zhengzhou		Zhumadian		Anyang		Zhengzhou		Zhumadian		Zhengzhou	
									E1	E2	E3	E4	E5	E6	E7	E8	E9	E10	E11	E12	E13	E14
Excalibur_c39508_88	3AL	AG	GG	58	101	50.2	48.3	TKW	0.026	0.016	0.143	0.567	0.042	0.036	0.022	0.002	0.158	0.158	0.048	0.091	0.085	0.091
						2.45	2.28	KWS	0.015	0.019	0.000	0.005	0.006	0.004	0.019	0.002	0.033	0.025	0.001	0.007	0.010	0.165
						19.3	18.9	FSNS	0.007	0.013	0.003	0.008	0.096	0.046	0.131	0.061	0.029	0.138	0.134	0.063	0.837	0.639
Excalibur_c19547_1012	4DL	AG	GG	67	92	1.6	1.8	SSNS	0.001	0.000	0.014	0.028	0.335	0.546	0.076	0.006	0.524	0.407	0.295	0.320	–	–
						2.6	2.3	FLBA	0.106	0.192	0.018	0.055	0.000	0.000	0.143	0.319	0.045	0.020	0.066	0.015	–	–
BS00060460_51	5BL	GG	TG	100	55	50.3	46.9	TKW	0.000	0.001	0.000	0.000	0.001	0.000	0.000	0.002	0.001	0.000	0.001	0.001	0.007	0.023
						2.4	2.3	KWS	0.000	0.008	0.013	0.002	0.087	0.236	0.017	0.090	0.015	0.010	0.038	0.019	0.138	0.184
						7.2	6.8	KL	0.000	0.000	0.000	0.000	0.000	0.000	0.000	0.000	0.000	0.000	0.000	0.000	0.000	0.000
Ra_c72517_981	2DL	AG	AA	102	54	2.4	2.2	KWS	0.789	0.389	0.024	0.116	0.021	0.018	0.002	0.011	0.025	0.113	0.010	0.009	0.008	0.035
						49.0	45.9	KNS	0.065	0.044	0.031	0.063	0.015	0.002	0.002	0.004	0.002	0.010	0.013	0.006	0.001	0.002
Jagger_c4951_122	5BL	TC	TT	69	93	47.5	50.2	TKW	0.000	0.003	0.001	0.003	0.009	0.002	0.001	0.018	0.022	0.014	0.002	0.003	0.081	0.039
						9.5	10.2	SL	0.000	0.000	0.000	0.000	0.001	0.001	0.007	0.004	0.000	0.003	0.000	0.001	0.000	0.001
						2.3	2.4	KWS	0.000	0.006	0.001	0.001	0.047	0.046	0.029	0.103	0.093	0.167	0.012	0.008	0.588	0.040

TKW, thousand kernel weight; KL, kernel length; KWS, kernel weight per spike; KNS, kernel number per spike; SL, spike length; FSNS, fertile spikelet number per spike; SSNS, sterile spikelet number per spike; FLBA, flag leaf base angle.

‘–’, indicated that data were missing.

### Utilization of superior alleles in wheat cultivars from the Yellow and Huai wheat region

**Figure 4:**
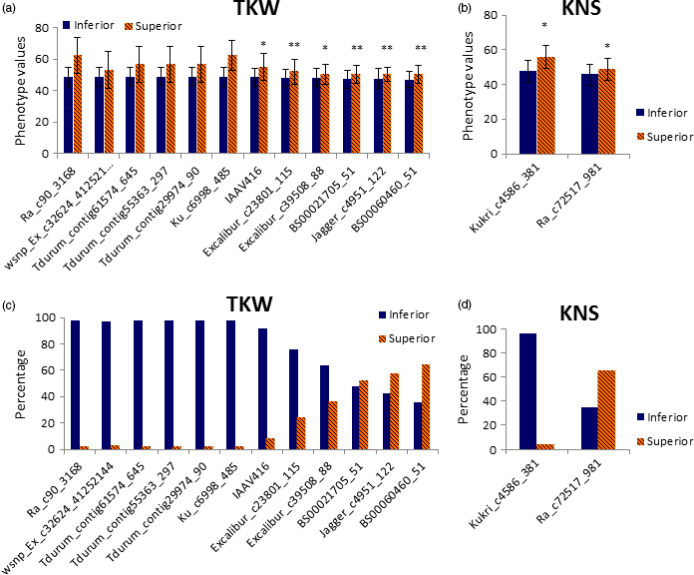
The phenotype values (a–b) and percentage of cultivars (c–d) with superior allele in different significant loci for TKW and KNS.

**Figure 5:**
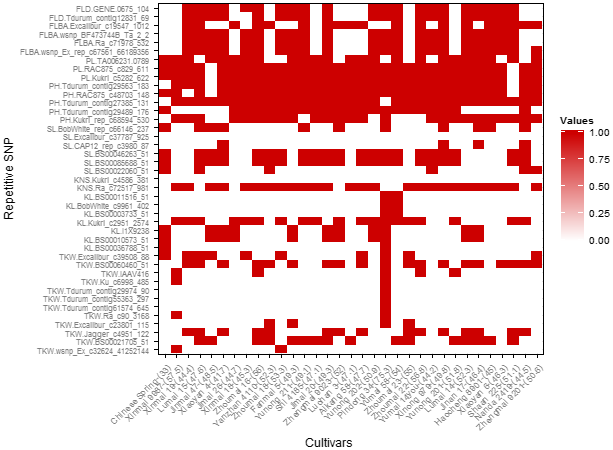
The superior allele loci distributions in popular cultivars. Red colours represent superior allele. The corresponding averaged TKW for each cultivar is in parentheses.

In this study, superior allele means regulating high TKW and KWS, long kernel length and spike length, more KNS and FSNS, short plant height and peduncle length and small FLBA and FLD. Based on repetivity and PVE of all significant SNPs in the 14 environments surveyed, some SNPs were considered as important genetic loci to possibly play the key role in modulating agronomic traits of wheat cultivars in the Yellow and Huai wheat region. For TKW, 12 genetic loci were selected to be important in the cultivars from this wheat region (Table 2). Cultivar Pindong 34 containing nine superior alleles at the 12 loci showed the averaged 75.3 g (ranged from 67.6 to 89.9 g) TKW over the 14 environments, whereas five cultivars with inferior alleles (with relatively lower TKW) at the 12 loci showed the averaged 39.4 g (ranged from 35.5 to 41.5 g) TKW (the averaged TKW of all cultivars surveyed was 49.0 g). Three of the 12 loci showed polymorphic in the two RIL populations (i.e. PC and UP). Physical mapping in the PC population indicated that the BS00021705_51 simultaneously regulated peduncle length and kernel length with PVEs of 7.1%–17.1% (Table 5; Figure S3), and the Excalibur_c39508_88 regulated spike length with PVE of 16.6%. Physical mapping in the UP population indicated that the Jagger_c4951_122 simultaneously regulated five agronomic traits, that is kernel width, germination index, germination energy, germination rate and germination rate in the first day (Table 5; Figure S3). It confirmed that these three SNPs were important genetic loci for agronomic traits. Distribution of allelic variation of these 12 loci indicated that three superior alleles at the BS00060460_51, Jagger_c4951_122 and BS00021705_51 loci showed more than percentages of 50% (Figure 4c), implying that the three loci had been widely taken advantage in increasing TKW of bread wheat. In addition, most of the 32 currently represented wheat cultivars (cumulative planting area >100 000 hectare) have also possessed superior alleles at these three loci (Figure 5). Therefore, it suggested that more attention should be paid to the remaining 9 loci in view of improvement of TKW in this wheat region. Of the nine loci, six loci (Table 2) were on the chromosome 6A. Cultivars with superior alleles at five of the six loci showed significantly higher TKW, ranging from 56.6 to 62.2 g, whereas cultivars with superior alleles of the remaining four loci showed the averaged TKW of between 50.2 and 52.9 g (Table S4; Figure 4a).

**Table 5:** QTL intervals and agronomic traits revealed by QTL mapping using candidate SNPs in the PC and UP populations

Population	Trait	Year and location	Chromosome	Position	Left marker	Right marker	LOD	PVE	Add
PC	SL	2015Anyang	3A	73	T210	Excalibur_c39508_88	3.0	13.6	‐0.4
		2015 Zhengzhou	3A	71	T210	Excalibur_c39508_88	5.5	19.5	‐0.6
	PL	2014 Anyang	6B	37	P446	BS00021705_51	3.94	6.1	‐1.6
		2014 Zhengzhou	6B	37	P446	BS00021705_51	3.2	8.1	‐1.7
	KNS	2015Anyang	6A	154	P362	Kukri_c2951_2574	4.8	13.8	‐28.4
	KL	2014 Anyang	6B	43	BS00021705_51	P146	2.9	17.1	‐0.13
UP	KW	2014 Anyang	5B	5	Jagger_c4951_122	wms118	3.2	11.5	0.2
	GR	2015 Anyang	5B	7	Jagger_c4951_122	wms118	13.8	1.2	0.1
	GR_1st	2015 Anyang	5B	11	Jagger_c4951_122	wms118	7.7	5.6	13.3
		2015 Zhengzhou	5B	5	Jagger_c4951_122	wms118	9.3	14.9	8.3
	GE	2015 Anyang	5B	7	Jagger_c4951_122	wms118	17.0	1.4	0.1
		2015 Zhengzhou	5B	5	Jagger_c4951_122	wms118	21.8	6.2	0.1
	GI	2015 Anyang	5B	7	Jagger_c4951_122	wms118	5.6	2.6	0.1
		2015 Zhengzhou	5B	3	Jagger_c4951_122	wms118	22.3	15.5	0.1

Position (cM), distance between QTL and the top marker of each linkage map; LOD, threshold of 2.5 was set for declaring the presence of QTL; PVE (%), phenotypic variation explained by QTL; GR, germination rate; GR_1st, germination rate in the 1st day; GI, germination index; GE; germination energy; Add, Positive ‘additive effect’ indicates an increasing effect from Proteo in the PC population, and UC1110 in the UP population; negative ‘additive effect’ indicates an increasing effect from Chaja in the PC population, and PI610750 in the UP population.

For kernel length, seven SNPs were selected as important genetic loci (Table 2). Physical mapping results in the PC population (Table 5; Figure S3) indicated that Kukri_c2951_2574 regulated KNS with PVE of 13.8%. Two cultivars (Ningchun 10 and Pindong 34) containing all superior alleles at the seven loci showed averaged 8.66 cm kernel length (ranged from 7.70 to 9.50 cm) in the 14 environments, whereas 50 cultivars without any superior allele showed the averaged 6.87 cm kernel length (ranged from 5.3 to 7.58 cm). Furthermore, cultivars with superior alleles at the three loci (BS00010573_51, IACX9238 and Kukri_c2951_2574) were very prevalent, and cultivars with the superior alleles at the remaining four loci were very rare (Table S4) as well as in the 32 currently represented wheat cultivars (Figure 5), suggesting that more attention should be paid to these four loci in view of improvement of kernel length in this region. In addition, BS00003733_51, BobWhite_c9961_402 and BS00011516_51 of the seven SNPs were located on 3A, 3B and 3D, respectively, indicating that they may be homologous.

For KNS, only two SNPs were selected as important genetic loci (Table 2). Further analysis indicated that superior allele of Ra_c72517_981 has played the important role in regulating KNS of wheat surveyed due to its high percentage of 65.4%. Kukri_c4586_381 was mapped to the QTL intervals P533~T64 on chromosome 6B in the PC population (Figure S3). Five cultivars containing superior alleles at the two loci showed an averaged KNS of 54.7 (ranged from 49.2 to 62.4), while 59 cultivars without superior allele showed the averaged KNS of 45.7 (ranged from 35.5 to 57.5). Cultivars with superior allele of Kukri_c4586_381 showed more KNS when compared to cultivars with superior allele of Ra_c72517_981 (Figure 4b). However, only seven cultivars were detected to have superior allele of Kukri_c4586_381 in the cultivars surveyed (Figure 4d), and all of the 32 currently popular cultivars mentioned above lack superior allele of Kukri_c4586_381 (Figure 5). It suggests that the Kukri_c4586_381 was not fully utilized so far and could be considered as an important genetic locus, especially in the currently represented wheat cultivars.

For spike length, six SNPs were selected as important genetic loci (Table 2). Seven cultivars containing five superior alleles at the six loci showed an averaged spike length of 11.7 cm (ranged from 8.2 to 15.1 cm), whereas 47 cultivars without superior allele showed the averaged spike length of 9.1 cm (ranged from 6.5 to 10.7 cm). Further analysis indicated that superior alleles at three loci (Table S4) prevalently presented in cultivars surveyed, and most of the 32 currently popular cultivars have contained loci BS00085688_51 and BS00046263_51 (Figure 5). However, superior alleles at the remaining three loci showed less than percentage of 14% in cultivars surveyed as well as currently popular cultivars, indicating that these three loci should be paid more attention in view of improvement of spike length.

For plant height and peduncle length, totally eight SNPs were selected as important genetic loci (Table 2). However, most of the cultivars have possessed superior alleles at three of the five plant height‐related SNP loci and two of the three peduncle length‐related SNP loci, and the remaining three loci should be paid more attention in view of decreasing plant height in this wheat region (Table S4).

For FLBA and FLD, totally six SNPs were selected as important genetic loci, and three of them simultaneously regulated FLBA and FLD (Tables 2, S4; Figure S4). All six SNPs loci were prevalent in the cultivars surveyed as well as the 32 currently popular wheat cultivars. However, there were 52 (31.9%) cultivars containing all superior alleles and 41 (25.2%) cultivars without any superior allele at the six loci, indicating that these six SNPs still could be taken advantage of compacting plant type.

### Distribution of superior alleles in bread cultivars of different stages in the Yellow and Huai wheat region

**Figure 6:**
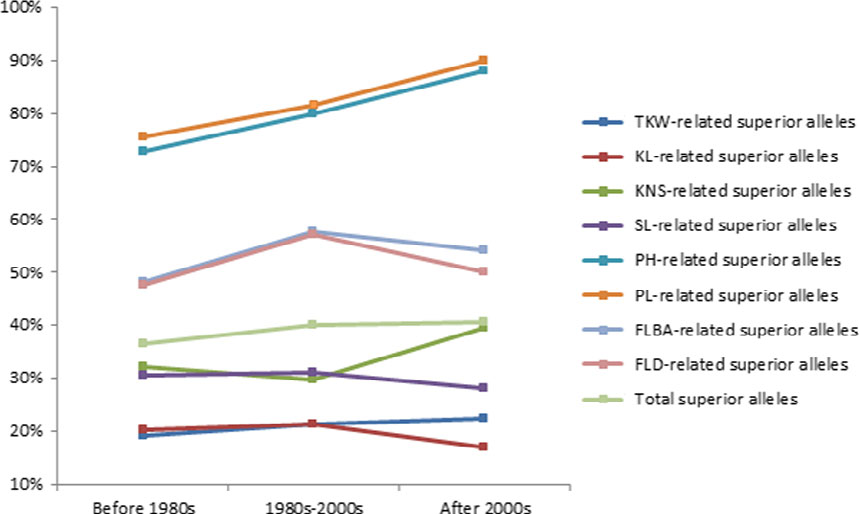
Percentages of superior alleles in cultivars released before 1980s, 1980s–2000s and after 2000s in the Yellow and Huai wheat region.

Based on released time, the natural population used in this study could be divided into three eras, that is before 1980s, 1980s–2000s and after 2000s, and their percentages were 41.1%, 30.1% and 28.8%, respectively. Analysis of distribution of superior alleles indicated that percentages of total superior alleles at the above‐selected 41 SNP loci are 36.6%, 39.9.0% and 40.6% in the cultivars of before 1980s, 1980s–2000s and after 2000s (Figure 6), respectively. Furthermore, percentages of superior alleles related to TKW, plant height and peduncle length gradually increased from before 1980s, 1980s–2000s to after 2000s (Figure 6). It suggested that superior alleles were getting popular with time in cultivars of the Yellow and Huai wheat region of China and modern cultivars had integrated many superior alleles (Figure 6), especially for peduncle length‐ and plant height‐related superior alleles. For example, all of the modern cultivars have possessed superior alleles at four SNP loci, that is Tdurum_contig27385_131, Tdurum_contig29563_183, Kukri_c5282_622 and RAC875_c829_611. However, there were still 19 of the 41 SNP loci showing less than percentages of 50% in modern cultivars (after 2000s; Figure S4), suggesting they should be paid more attention in view of improvement of yield‐related traits in cultivars of the Yellow and Huai wheat region of China.

## Discussion

### Population structure and linkage disequilibrium analysis

LD in population is the foundation of GWAS, whereas it always affected by genetic draft, population stratification and natural selection. Of the above threats, population stratification is recognized as a major one to the validity of GWAS results (Cardon and Palmer, 2003). STRUCTURE software and PCA analysis obtained same results about the population structure. The quantile–quantile (Q–Q) plots (Figure S2) indicated the influence of population stratification was controlled reasonably in the selected cultivars. Linkage disequilibrium decay rate varied among different mapping populations. We observed highest linkage disequilibrium in the A genome, whereas Sukumaran *et al*. (2015) found highest linkage disequilibrium in D genome, and Ain *et al*. (2015) observed highest linkage disequilibrium in B genome.

### Marker–trait association and QTL mapping

#### Kernel size‐related traits

Previous findings suggested that genes associated with kernel size‐related traits were mainly distributed on chromosomes 2A (Hou *et al*., 2014; Ma *et al*., 2012), 2B and 2D (Hou *et al*., 2014), 3A (Wang *et al*., 2015), 3D (Wang *et al*., 2015; Zhang *et al*., 2011) and 6A (Bednarek *et al*., 2012), specifically *TaGW2* on chromosome 6A and *TaCKX2* on chromosome 3D regulating grain characteristics. Related QTLs were mapped to almost all the chromosome regions. They were identified on chromosome 2B, 2DS, 4A, 4B, 5A, 7AL, 7B and 7DL for grain yield (Maccaferri *et al*., 2008; Narasimhamoorthy *et al*., 2006; Peleg *et al*., 2011; Quarrie *et al*., 2006), on chromosomes 1A, 4B, 5A and 7D for kernel length, width and weight (Gegas *et al*., 2010; Mir *et al*., 2012), on chromosomes 2A, 2B, 2D, 3B, 4B, 4D, 5A, 5B, 6A, 7A and 7B for TKW (Bednarek *et al*., 2012; Narasimhamoorthy *et al*., 2006; Peleg *et al*., 2011).

In this study, SNPs significantly associated with TKW were mainly observed on chromosomes 7AS, 6AS, 6AL, 6BS, 5BL, 3AL and 3DL. BS00021705_51 on chromosome 6BS identified in 12 of 14 environments was also mapped to two QTL intervals (P446~BS00021705_51 and BS00021705_51~P146) in PC population, regulating peduncle length and kernel length, which indicated this SNP may be significantly linked to a new yield‐related genetic locus on chromosome 6BS. BS00060460_51 and Jagger_c4951_122 on chromosome 5BL were located in a pleiotropic region, affecting KWS, kernel length and spike length. QTL mapping results in UP population in the current study also indicated that Jagger_c4951_122 was located in a pleiotropic QTL interval (Jagger_c4951_122~wms118) affecting five new agronomic traits, including kernel width, germination index, germination energy, germination rate and germination rate in the 1st day. A positive ‘additive effect’ indicated that an increasing effect derived from cv UC1110 with allele T and cultivars with allele TT in Jagger_c4951_122 also showed higher TKW in the association population, which was consistent with the GWAS results. Besides, Ain *et al*. (2015) also detected a pleiotropic region (wsnp_Ex_rep_c66651_64962429~TA002629‐0202) on chromosome 5BL affecting TKW and plant height. A similar study reported chromosome region that affects kernel yield and TKW in CIMMYT spring wheat lines was also on the chromosome 5B (73 cM) (Edae *et al*., 2014). Therefore, BS00060460_51 and Jagger_c4951_122 on chromosome 5BL may be newly identified genetic loci, and may play an important role in regulating kernel yield in the Yellow and Huai winter wheat region.

Another pleiotropic SNP Excalibur_c39508_88 affecting TKW, KWS, kernel width and FSNS was revealed on chromosome 3AL. It was mapped to a QTL interval (T210~Excalibur_c39508_88), affecting spike length in the PC population consistently in two environments. A negative ‘additive effect’ indicated that an increasing effect derived from cv Chaja with allele A and cultivars with allele AG in Excalibur_c39508_88 also showed higher TKW, KWS and more FSNS in the association population. This SNP may also closely link to genetic locus on chromosome 3A. Earlier studies reported a QTL for yield in chromosome 6AS in winter wheat (Snape *et al*., 2007) and spring wheat (Lopes *et al*., 2014); in this study, we also consistently detected wsnp_Ex_c32624_41252144 on chromosome 6AS affecting TKW explaining 24% of the phenotypic variation in 13 of the 14 environments.

Gao *et al*. (2015) conducted a biparental QTL mapping using the cross of Zhou8425B/Chinese Spring and mapped 13 QTLs for TKW, in which four SNPs on chromosome 6AS and one SNP on chromosome 6AL were overlapped with the current GWAS results. Ra_c90_3168 on 6AL explaining 24.1% of the phenotypic variation identified in six environments showed a positive effect on TKW. The remaining four SNPs on 6AS (Ku_c6998_485, Excalibur_c33017_141, Ku_c9204_918, Ku_c935_3041 explaining 25.9%, 27.9%, 28.2%, 28.2% of the phenotypic variation) were detected in five, three, three and three environments, respectively. Ku_c9204_918 and Ku_c935_3041 were positively associated with TKW, while Ku_c6998_485 and Excalibur_c33017_141 were negatively associated with TKW. Furthermore, Gao *et al*. (2015) also detected this QTL (Ku_c32392_967~wsnp_RFL_Contig2523_2130662) in all of the surveyed four environments with PVE of 7.3%, 4.8%, 9.9%, and 9.2%, respectively. These associated markers on chromosome 6A may closely link to *TaGW2* regulating kernel weight, but more work will be needed to confirm.

Repetitive SNPs associated with kernel length were consistently identified on chromosomes 7BS, 6AS, 5AL, 5BL, 4AS, 3AS, 3B, 3DS and 1AL, in which chromosomes 1A, 3A, 4B, 5A and 6A corresponded to previously mapped QTL regions (Gegas *et al*., 2010). Kukri_c2951_2574 on chromosome 6A was mapped to a QTL interval (P362~Kukri_c2951_2574) in the PC population affecting KNS, explaining 13.8% of the phenotypic variation. BS00011516_51 on chromosome 3DS, consistently identified in seven environments, may closely link to *TaCKX2* on chromosome 3D regulating grain characteristics. Kukri_c2951_2574 on chromosome 6AS, which was consistently identified in seven environments, may be a new identified genetic locus.

Poor repeatability in significant SNPs associated with kernel width may indicate that there was a large distance between SNPs and genetic loci affecting kernel width. Gegas *et al*. (2010) also pointed out that modern hexaploid wheat has significantly reduced kernel shape variation compared to ancestral wheat species who retained a relatively large percentage of the nucleotide diversity of A and B genomes. We remeasured the kernel diameter by the SKCS and associated three new repetitive SNPs.

### Plant architecture‐related traits

In plant architecture‐related traits, plant height is a key factor regulating kernel yield. The well‐known *Rht* genes *Rht‐B1b*,* Rht‐D1b*,* Rht4*,* Rht5*,* Rht8*,* Rht9*,* Rht12* and *Rht13* were located on chromosomes 4BS, 4DS, 2BL, 3BS, 2DS, 5AL, 5AL and 7BS, respectively (Ellis *et al*., 2005). In the current study, repetitive SNPs linked to plant height were consistently identified on chromosomes 7DS, 6DL, 5AL, 4DS, 4BS, 2DS, 2BS, 2AS and 1BL. Stable marker Kukri_rep_c68594_530, with averaged PVE of about 59%, and consistently identified in 13 of 14 environments, was located on chromosome 4DS. Chen *et al*. (2015) also detected Kukri_rep_c68594_530 associated with plant height in Chinese wheat in one of four investigated environments. In the study by Gao *et al*. (2015), one QTL *QPH.caas‐4D* was detected associated with plant height using a RIL population derived from the cross between Zhou 8425B and Chinese Spring investigated under limited and full irrigation environments, and the authors assumed it to be the *Rht‐D1b* locus. It is possible that Kukri_rep_c68594_530 is closely linked to the *Rht‐D1b* gene.

Tdurum_contig29563_183 on chromosome 4BS, consistently detected in seven environments, with averaged PVE of 50.4%, is possibly linked to the *Rht‐B1b* gene given they have the same chromosome location. Gao *et al*. (2015) also repetitively detected two QTLs on 4BS and one QTL on 4DS associated with plant height in a biparental population using the 90K SNP assay. RAC875_c48703_148 on chromosome 2DS, with averaged PVE of 54.3%, and consistently identified in 11 of 14 environments, may be linked to the *Rht8* gene given they have the same chromosome location. Previous studies indicated that the *Rht‐B1b*,* Rht‐D1b* and *Rht8* genes were widely distributed and utilized for breeding programme in the Yellow and Huai wheat region (Yang *et al*., 2006). Tdurum_contig29489_176 on chromosome 6DL with 55.8% of PVE and Tdurum_contig27385_131 on chromosome 1BL with 55.4% of PVE, significantly associated with plant height in 11 of 14 environments in this study, were possibly two new plant height‐related sites and could be further used for improvement of agronomic traits in Chinese wheat breeding programme. Sukumaran *et al*. (2015) and Ain *et al*. (2015) also detected significant marker linked to plant height on chromosomes 1A, 6A and 2B. However, no significant association was detected for plant height on chromosome 4B or 4D.

QTLs linked to flag leaf characters were mainly mapped to chromosomes 1BS, 1BL, 2BL, 3BL, 3AL and 5B for flag leaf length and to chromosomes 2DL, 3BL, 5BS, 6A and 7AS for flag leaf width (Edae *et al*., 2014). However, FLBA and FLD investigated in this study were not focused. Repetitive SNPs Tdurum_contig12831_69 on chromosome 2DL consistently identified in eight environments, and wsnp_Ex_rep_c67561_66189356, wsnp_BF473744B_Ta_2_2 and Ra_c71978_532 on chromosomes 2BL, 2BL and 5AL were consistently identified in nine environments. These newly identified SNPs were colocalized in FLBA and FLD, and deserved to be further analysed.

### Superior alleles for pyramid breeding

Uncovering superior allele loci was beneficial to pyramid breeding. For example, Pindong 34 that possesses nine superior allele of the 12 TKW‐related loci, resulting in an extremely high TKW in 14 environments. We compared the averaged TKW regulated by superior and inferior alleles at three loci (i.e. BS00021705_51, Jagger_c4951_122, Excalibur_c39508_88) and found that all of the phenotype differences reached extremely significant level when integrating three superior alleles, which provided a guidance for multilocus pyramid breeding (Table S5).

Popular wheat cultivars have always been the major contributors to wheat yield in the Yellow and Huai wheat region. Most of the 32 selected popular cultivars had possessed superior alleles at the loci BS00060460_51, Jagger_c4951_122 and BS00021705_51 for TKW, Kukri_c2951_2574 for kernel length, Ra_c72517_981 for KNS, BS00085688_51 and BS00046263_51 for spike length, Tdurum_contig27385_131, RAC875_c48703_148, Tdurum_contig29563_183, Kukri_c5282_622 and RAC875_c829_611 for plant height and peduncle length (Figure 5). However, not all superior alleles were found in significant loci, especially for loci associated with TKW. Superior alleles of Kukri_c4586_381 for KNS are not detected in any of the 32 popular wheat cultivars. Cultivar Pindong 34 possessing nine superior alleles of the 12 TKW‐related loci for TKW showed averaged 75.3 g TKW in the 14 surveyed environments, while the averaged TKW in the natural population was 49.0 g. Chinese Spring with one superior allele of the 12 loci showed only the average 33 g TKW. Superior alleles for TKW are mainly detected in cultivars Zhoumai 23, Zhoumai 16, Yanzhan 4110, Xinmai 9987, Zhoumai 22 and Shaan 225 at loci BS00060460_51, Jagger_c4951_122 and BS00021705_51. However, three Chinese cultivars Zhoumai 18, Zhoumai 22 and Xiaoyan 6 also showed high improvement potential at the loci BS00021705_51, Excalibur_c23801_115, Jagger_c4951_122, Ra_c90_3168 and Ku_c6998_485, as compared with Pindong 34, despite of their great contribution for wheat yield in the Yellow and Huai wheat region. In addition, superior allele of Kukri_c4586_381 for KNS deserves to be utilized to improve kernel numbers.

Additionally, Chinese wheat breeders would like to select cultivars with short plant height, peduncle length and small FLBA to get more spike number in unit area. Therefore, three plant height‐related loci (Kukri_rep_c68594_530, Tdurum_contig29489_176 and TA006231‐0789) and six FLBA‐related and FLD‐related loci (wsnp_Ex_rep_c67561_66189356, Ra_c71978_532, wsnp_BF473744B_Ta_2_2, Excalibur_c19547_1012, GENE‐0675_104 and Tdurum_contig12831_69) will be helpful for decrease in plant height and compact plant type in most cultivars from the Yellow and Huai wheat region. In summary, to increase wheat yield, superior alleles for yield‐related traits should be integrated properly by marker‐assisted selection for multiloci pyramid breeding.

## Materials and methods

### Plant materials

Based on their pedigree, released regions, agronomic performance, importance (backbone parents or not) and cultivated area, a total of 163 bread wheat cultivars collected from the Yellow and Huai Valley of China, including Henan, Hebei, Shaanxi, Shanxi and Shandong provinces, were selected as association panel (Chen *et al*., 2013; Wang *et al*., 2015). These cultivars were planted and harvested in 2012–2013, 2013–2014 and 2014–2015. In each cropping season, all the surveyed cultivars were planted in Zhengzhou Scientific Research and Education Center of Henan Agricultural University (34.8703°N, 113.5975°E), Anyang Academy of Agricultural Science (36.0960°N, 114.4117°E) and Zhumadian Academy of Agricultural Science (33.0117°N, 114.0522°E), respectively. The field experiment was performed using a completely randomized design. Each plot contained four 200‐cm‐long rows with 23 cm between neighbouring rows and 10 cm between neighbouring plants. All of the cultivars surveyed were, respectively, planted at two land parcels in each location.

Two F_10_ RIL populations UC1110 × PI610750 (UP) with 187 lines and Proteo × Chaja (PC) with 97 lines were used to validate significant SNPs revealed by GWAS. The two RIL populations grew well at Anyang and Zhengzhou in the 2013–2014 and 2014–2015 cropping seasons. The field experiments were designed as mentioned above.

### Phenotyping

For each cultivar surveyed, ten spikes from ten different single plants were marked with red ribbon and were used to investigate agronomic traits including plant height, spike length, peduncle length (PL), fertile spikelet number per spike (FSNS), sterile spikelet number per spike (SSNS), total spikelet number per spike (TSNS), flag leaf base angle (FLBA) and flag leaf direction (FLD) before harvest. Plant height was measured after physiological maturity by measuring the distance between the stem base and the top of the spike excluding awns. Flag leaf base angle means the angle between the wheat stem and the flag leaf base and was divided into four hierarchical levels with angle ranges of 0°–45°, 45°–90°, 90°–135° and 135°–180°. Flag leaf direction means the angle between the wheat stem and the flag leaf tip and was labelled as 1 for up and 2 for down. Other traits of the above‐mentioned ten marked spikes were further investigated after harvest, including kernel weight per spike, kernel number per spike, kernel length, kernel width and thousand kernel weight. Kernel length and kernel width were calculated from five replications of ten random kernels. TKW was measured by weighing 500 randomly selected kernels from each plot. For the UP and PC populations, except for the 13 agronomic traits, we also investigated germination‐related traits, including germination rate (GR), germination rate in the 1st day (GR_1st), germination energy (GE: germination rate in the third day) and germination index (GI).

Kernel diameter was measured by the Perten Single Kernel Characterization System (SKCS) 4100, following the manufacturer's operation procedure (Perten Instruments North America Inc., Springfield, IL), using 300 random kernels. This system can calculate the average kernel diameter automatically.

### Genotyping and filtering

Genotyping was performed by Beijing Compass Technology & investment Co. Ltd (http://www.bjcompass.com/), using the 90K wheat genotyping assay (Wang *et al*., 2014), following the manufacturer's protocol. A quality preprocessing of genotyping data was done for sample call rate, SNP call rate, minor allele frequency (MAF) and Hardy–Weinberg equilibrium (HWE). This preprocessing was implemented in PLINK software (http://pngu.mgh.harvard.edu/purcell/plink/) (Purcell *et al*., 2007).

### Linkage disequilibrium and population structure analysis

Linkage disequilibrium (LD) among markers was calculated for the A, B and D genomes in the PLINK software. The window size for linkage disequilibrium calculation was set based on the number of SNPs located in each genome. Pair‐wise linkage disequilibrium was measured using the squared allele frequency correlations, according to Weir (Williams, 1996), and assessed by calculating *r* ^2^ for pairs of SNP loci.

Population structure of the collected 163 cultivars was assessed with unlinked markers (*r* ^2^ = 0) using STRUCTURE software 2.3.4 (Pritchard *et al*., 2000). A burn‐in of 1000 iterations followed by 1000 Monte Carlo Markov Chain (MCMC) replicates was implemented to estimate the number of subpopulations (*k*) in a putative range of 1–10. To estimate the robustness of inferred population structure, ten replications were conducted for each *k*. The subpopulation number was estimated using an *ad hoc* statistic delta *k* based on the rate of change in log probability of data between successive values (Evanno *et al*., 2005). Principle component analysis (PCA) was also conducted in R software for assessing the population structure and compared to the result of STRUCTURE.

### Genome‐wide association study

GWAS were implemented by GAPIT packages (Lipka *et al*., 2012) in R software, using the mixed linear model (PCA + K) (Yu *et al*., 2006; Zhang *et al*., 2010), which took the population structure and relative kinship into account. The variance–covariance kinship matrix (K) was automatically calculated using the VanRaden method (Vanraden, 2008). The first four principal components of the SNP data were included in the GWAS model. The threshold for *P* value was calculated based on the number of the markers (*P *= 1/*n*,* n* = total SNP used) according to the method of Li *et al*. (2013). To combine the GWAS results in all of the 14 environments, a uniform suggestive genome‐wide significance threshold (*P* value = 1.0e−3) was given.

### Candidate gene sites sequencing and analysis

Candidate gene sites were aligned and downloaded from the ViroBLAST database (https://urgi.versailles.inra.fr/blast/docs/aboutviroblast.html). Genome‐specific primers were designed using Primer3 (http://primer3.ut.ee/; Table S1). Genomic DNA was extracted from three pulverized kernels for each line of the two RIL populations, following the protocol of Chen *et al*. (2011). Two PCR products of each cultivar or line were sequenced from both directions by Shanghai Sangon Biotech Co., Ltd, using the Sanger method, and checked in Chromas 2.5.1 software (http://technelysium.com.au/wp/chromas/). Multiple alignments of the candidate sequences were performed using DNAMAN software with default parameters (version 8.0, Lynnon Corp., Quebec, Canada).

### QTL mapping

The genetic linkage map of the UP population previously constructed by Lowe *et al*. (2011) was composed of 1494 polymorphic probes (SSRs, DArTs and ESTs) mapped to 558 unique loci. The genetic linkage map of the PC population was composed of 2810 SNP polymorphic markers mapped to 767 unique loci using the 9K iSelect Beadchip Assay (Cavanagh *et al*., 2013). The two genetic linkage maps of the two RIL populations were used to map the candidate SNPs discovered in the 90K SNP genotyping assay.

Genetic linkage maps were constructed and associated SNPs were mapped to the two RIL populations, using the inclusive composite interval mapping (ICIM) method (Li *et al*., 2007) by the software IciMapping 4.0 (http://www.isbreeding.net/). In ICIM, the background markers were selected only once using the standard stepwise regression by considering all marker information simultaneously, and the phenotypic values were then adjusted by all markers retained in the regression equation except for the two markers flanking the current mapping interval. The adjusted phenotypic values were finally used for interval mapping. ICIM‐ADD model was selected for QTL mapping, and other parameters used the default designs. The threshold of LOD score was set as 2.5 for declaring the presence of QTL.

## Conflict of interest

The authors declare no conflict of interest.

## Author contributions

F.C. designed the project. C.S., F.Z., X.Z. and Z.D. investigated agronomic traits. C.S. and F.C. wrote the manuscript. C.S., F.C., F.Z. and D.C. performed the SNP sequencing experiments and computational analyses. C.S., X.Y. and X.Z. performed QTL mapping.

## Supporting information


**Figure S1** Frequency distribution of phenotypic variation of investigated agronomic traits in three locations over three years.

**Figure S2** Manhattan and quantile–quantile (Q‐Q) plots for 13 agronomic traits.

**Figure S3** Genetic linkage maps of the two RIL populations. (a‐c) Linkage maps for chromosome 3A, 6A and 6B in the PC population; (d) linkage map for chromosome 5B in the UP population.

**Figure S4** The superior allele loci distributions in the surveyed natural population. Red colours represent superior allele.

**Table S1** Primers used for detecting significant SNP sites in two RIL populations.

**Table S2** Summary of SNPs significantly associated with 13 agronomic traits.

**Table S3** Significant SNP numbers in pairwise traits.

**Table S4** The distribution of focused SNPs superior alleles in the natural population.

**Table S5** P value of t‐test for averaged TKW grouped by taking different number of superior alleles.
