# Supplementary material for: Genome‐wide association study for 13 agronomic traits reveals distribution of superior alleles in bread wheat from the Yellow and Huai Valley of China
Source: Plant Biotechnol J. 2017 Mar 2;15(8):953–69. doi: 10.1111/pbi.12690 (PMC5506658; doi:10.1111/pbi.12690)
Supplement: Supplementary file 1 — Figure S1 Frequency distribution of phenotypic variation of investigated agronomic traits in three locations over three years. [file PBI-15-953-s004.pdf]

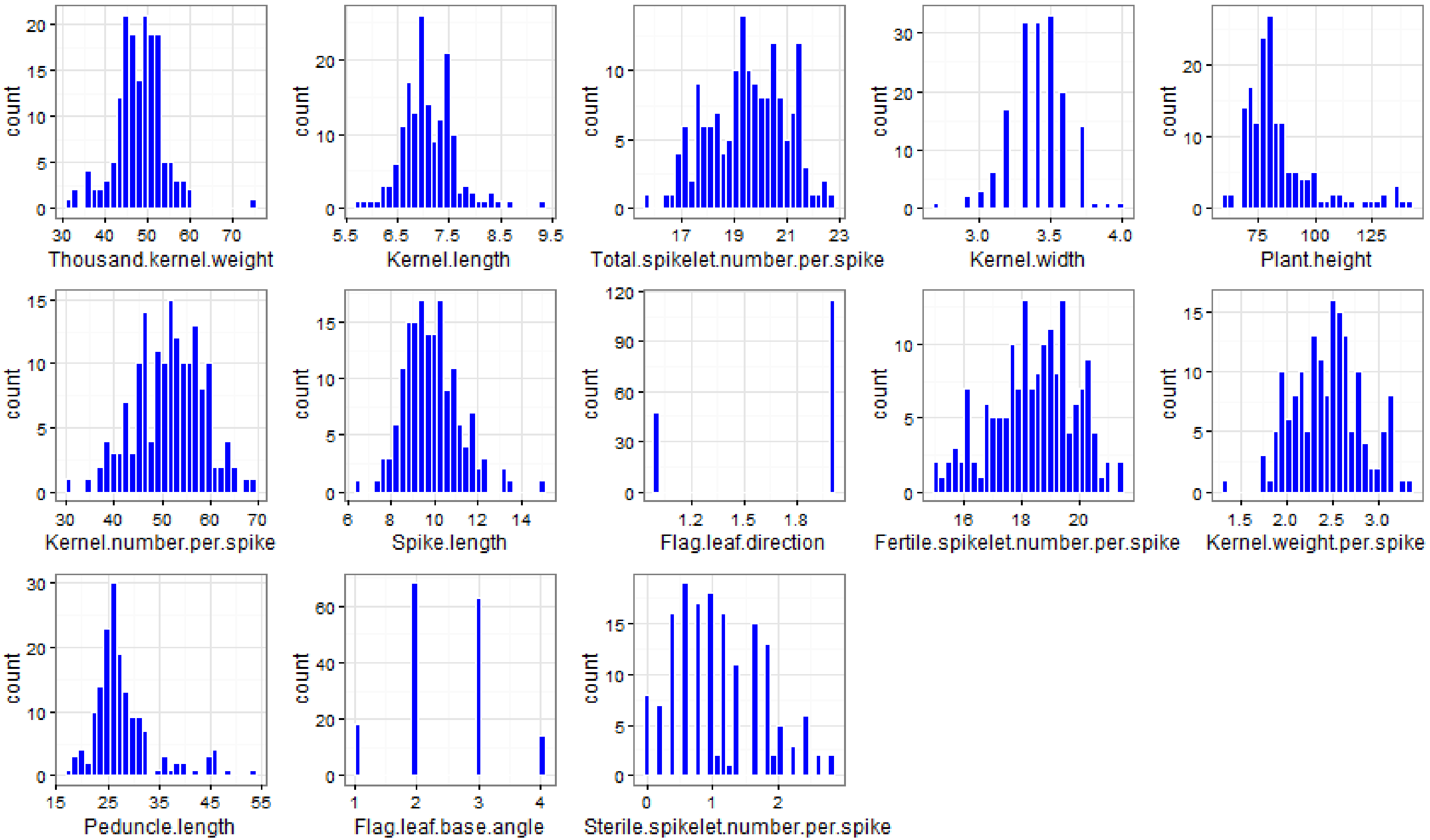

Fig. S1-1 Frequency distribution of phenotypic variation of investigated agronomic traits in 2013, Anyang, environment 1.

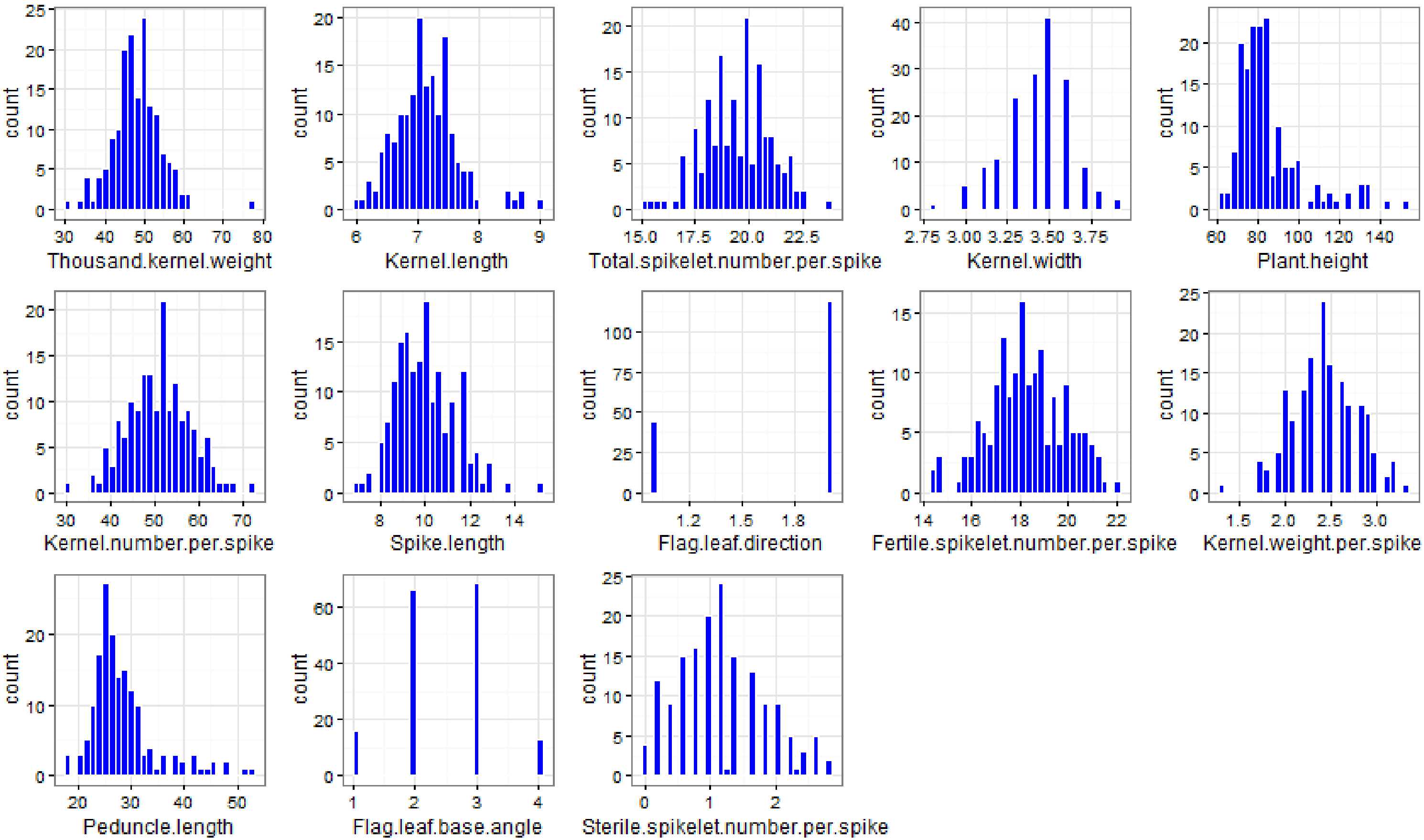

Fig. S1-2 Frequency distribution of phenotypic variation of investigated agronomic traits in 2013, Anyang, environment 2.

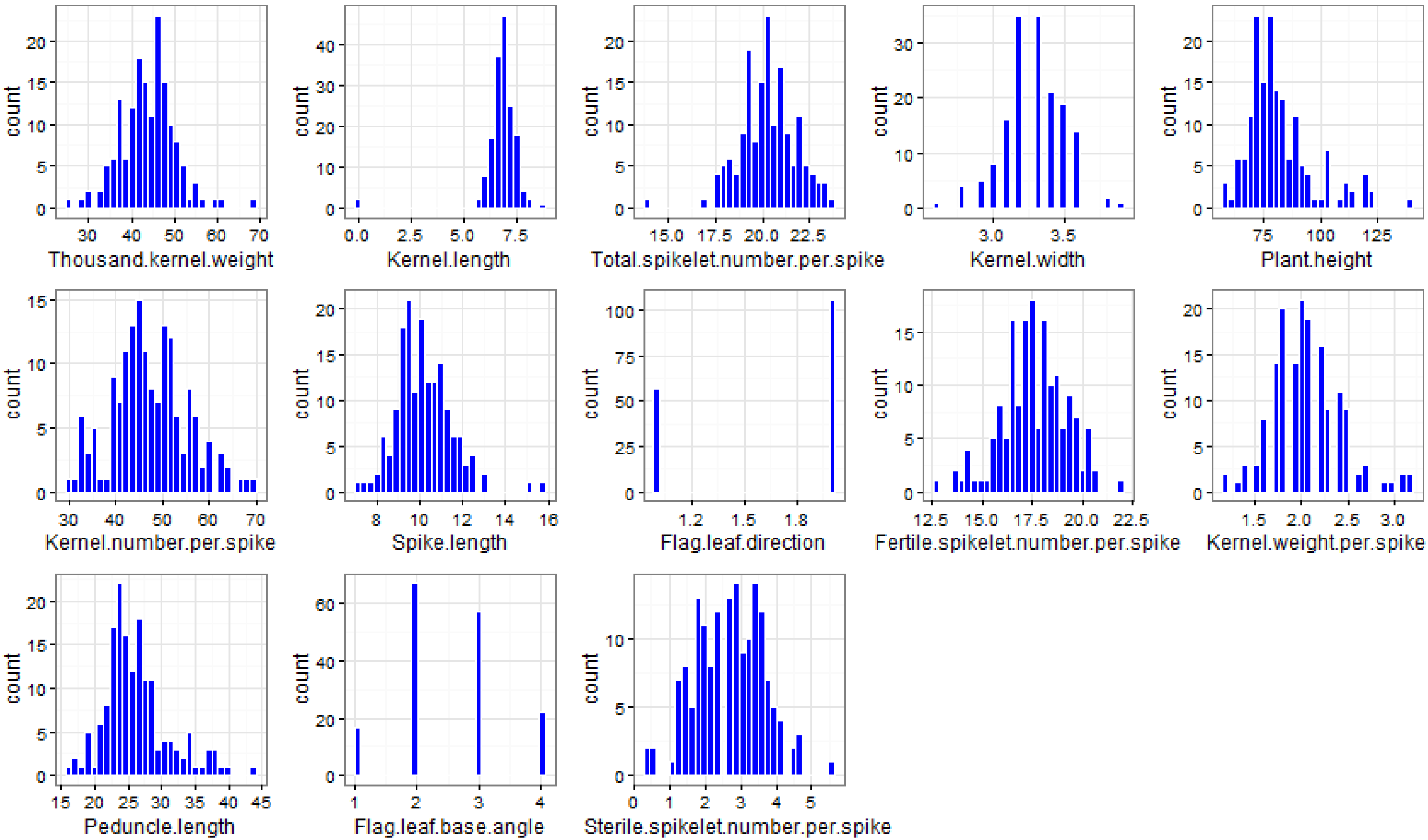

Fig. S1-3 Frequency distribution of phenotypic variation of investigated agronomic traits in 2013, Zhengzhou, environment 3.

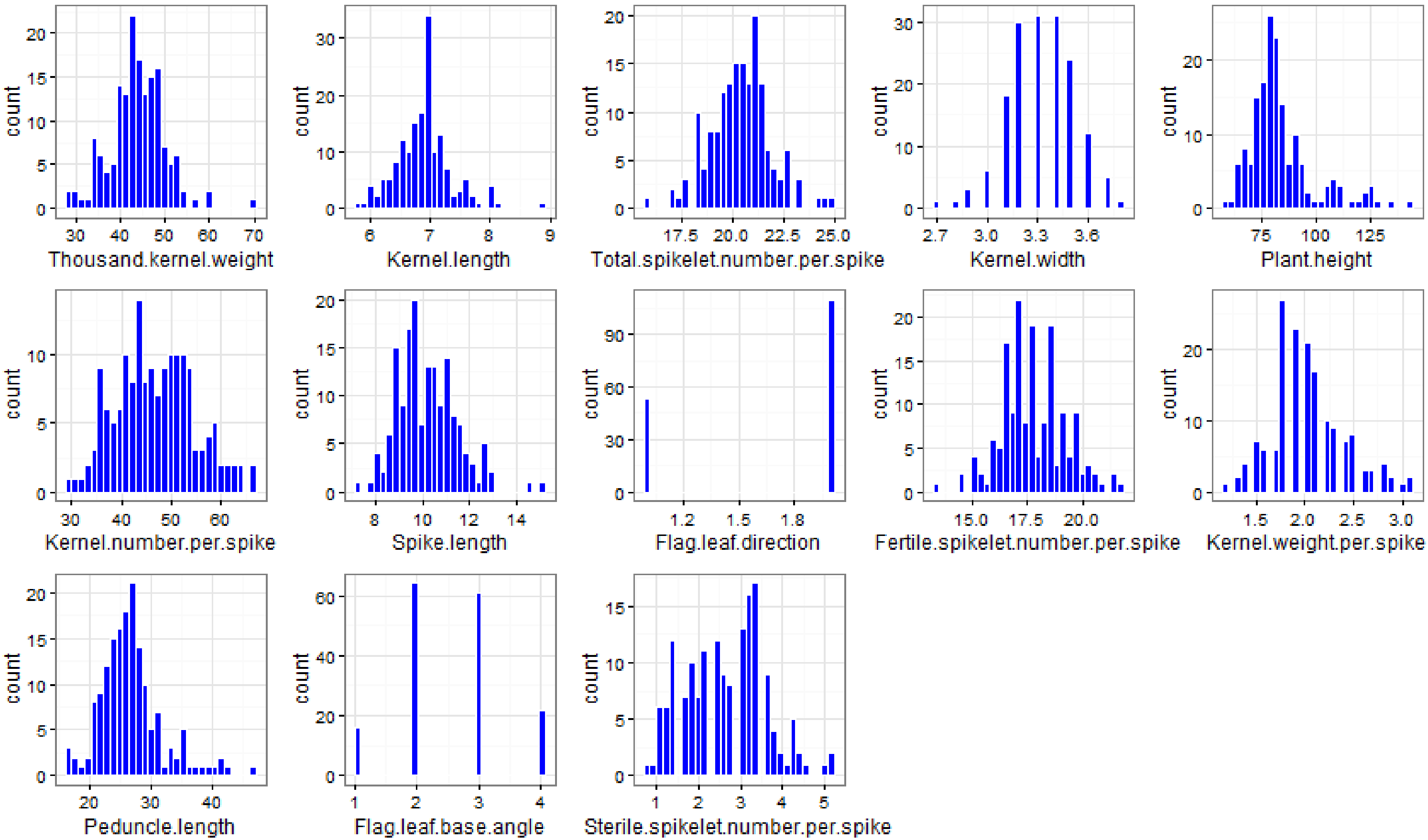

Fig. S1-4 Frequency distribution of phenotypic variation of investigated agronomic traits in 2013, Zhengzhou, environment 4.

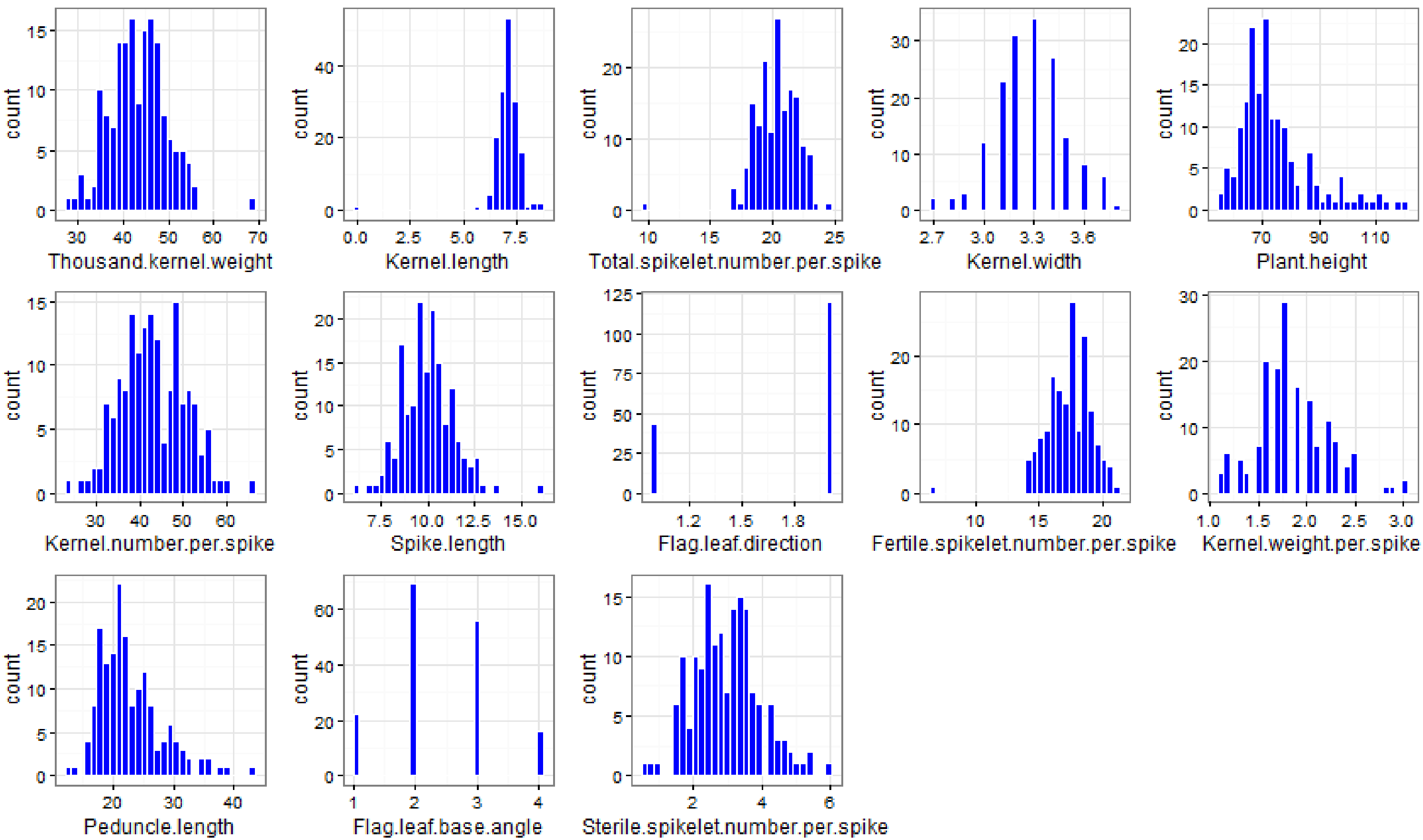

Fig. S1-5 Frequency distribution of phenotypic variation of investigated agronomic traits in 2013, Zhumadian, environment 5.

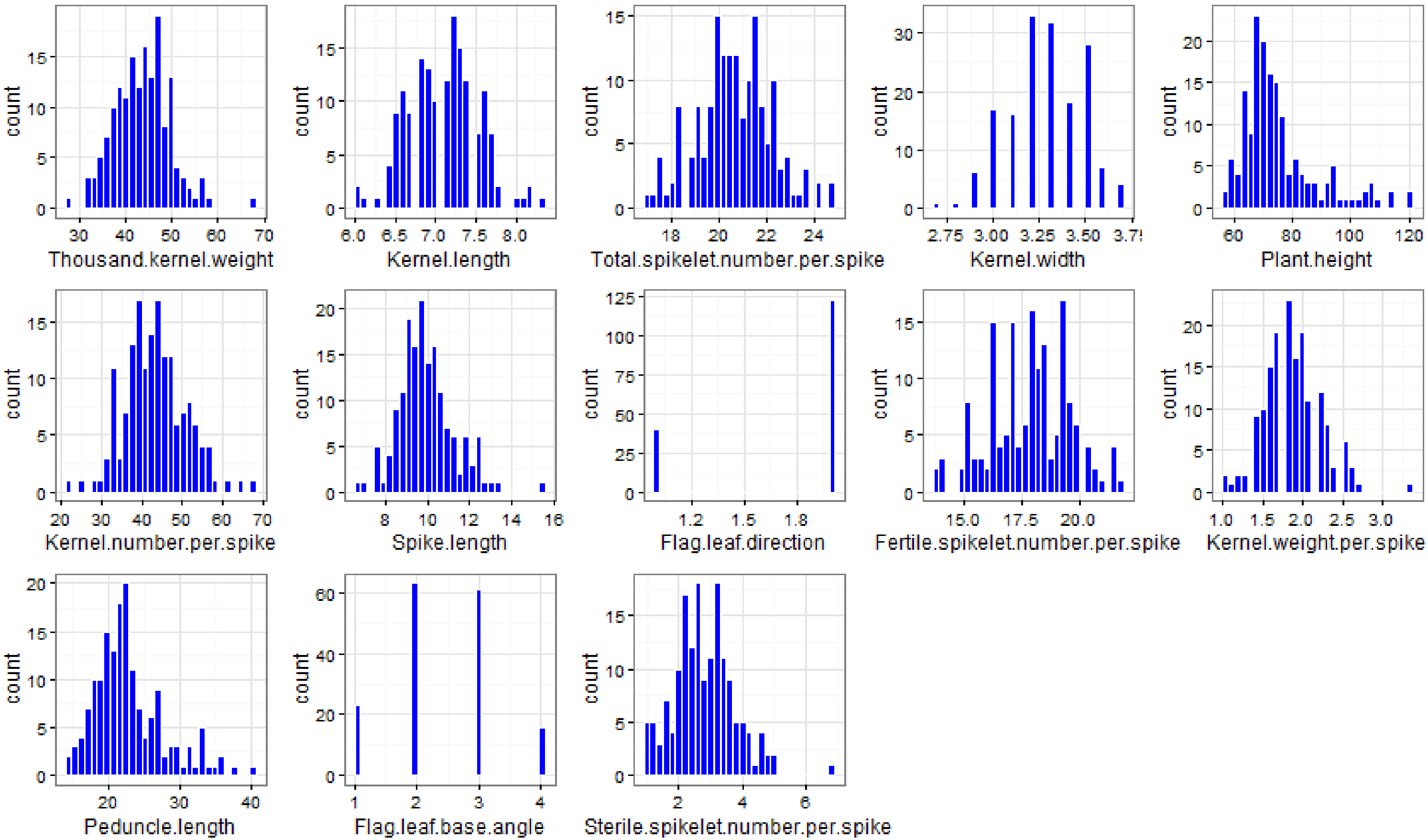

Fig. S1-6 Frequency distribution of phenotypic variation of investigated agronomic traits in 2013, Zhumadian, environment 6.

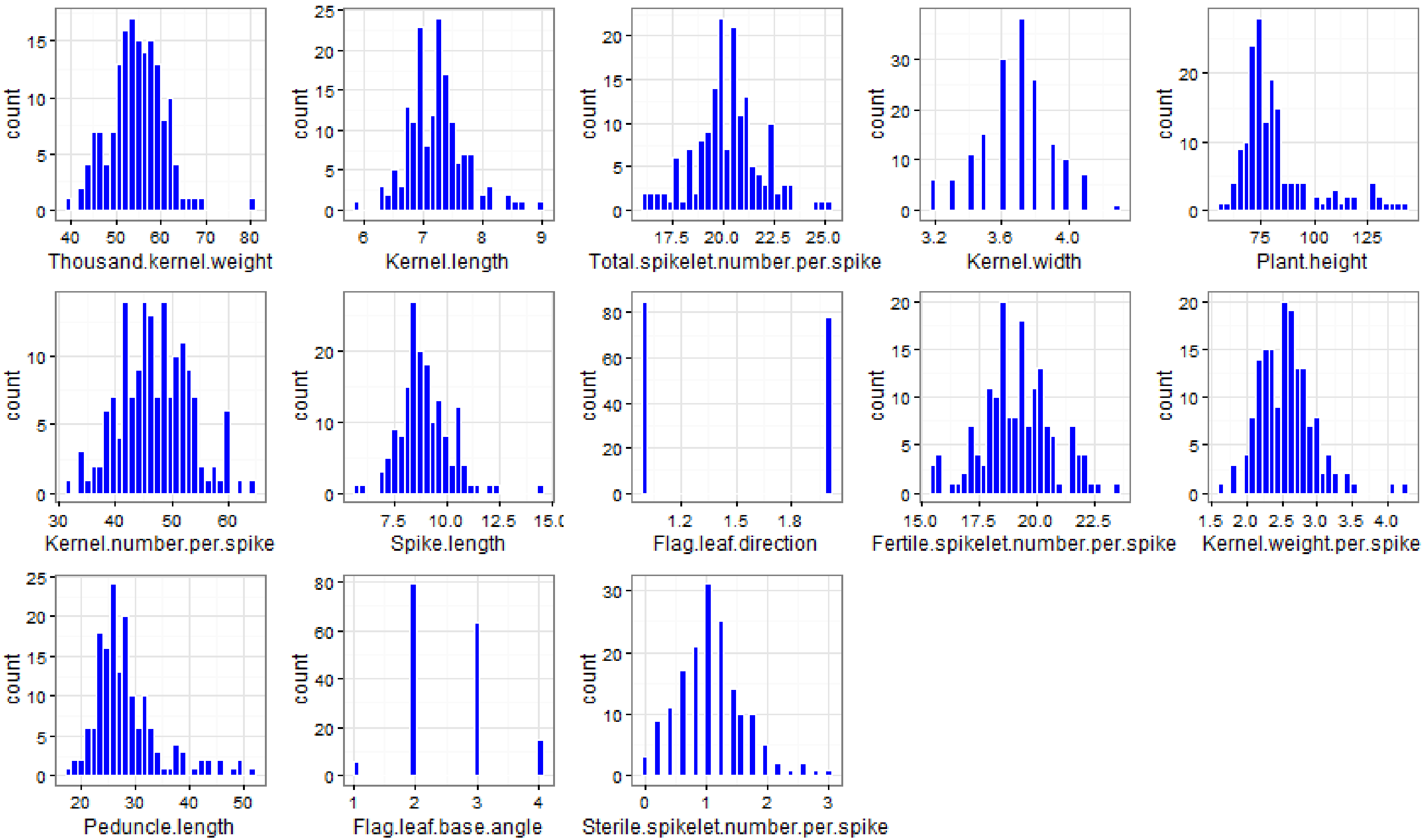

Fig. S1-7 Frequency distribution of phenotypic variation of investigated agronomic traits in 2014, Anyang, environment 7.

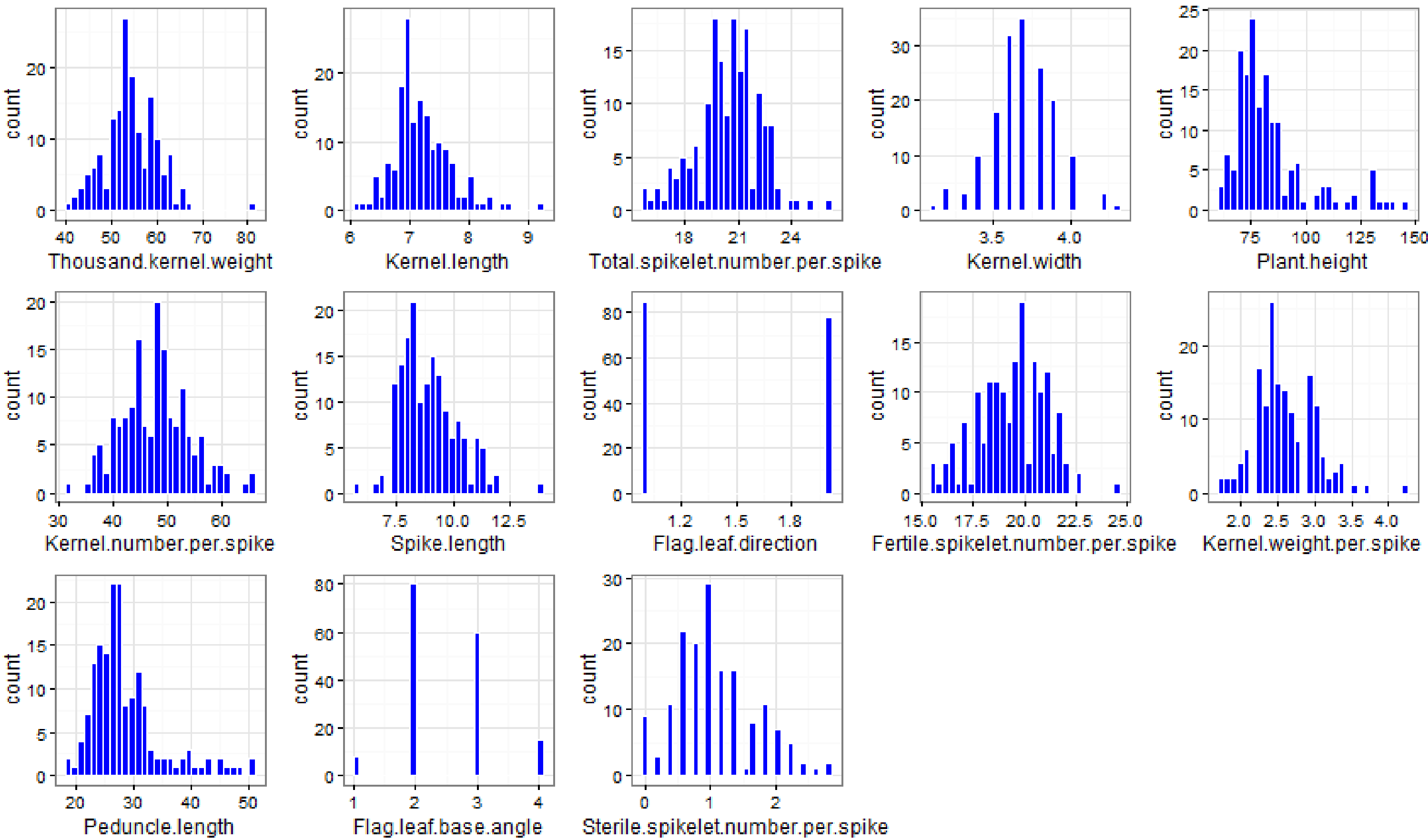

Fig. S1-8 Frequency distribution of phenotypic variation of investigated agronomic traits in 2014, Anyang, environment 8.

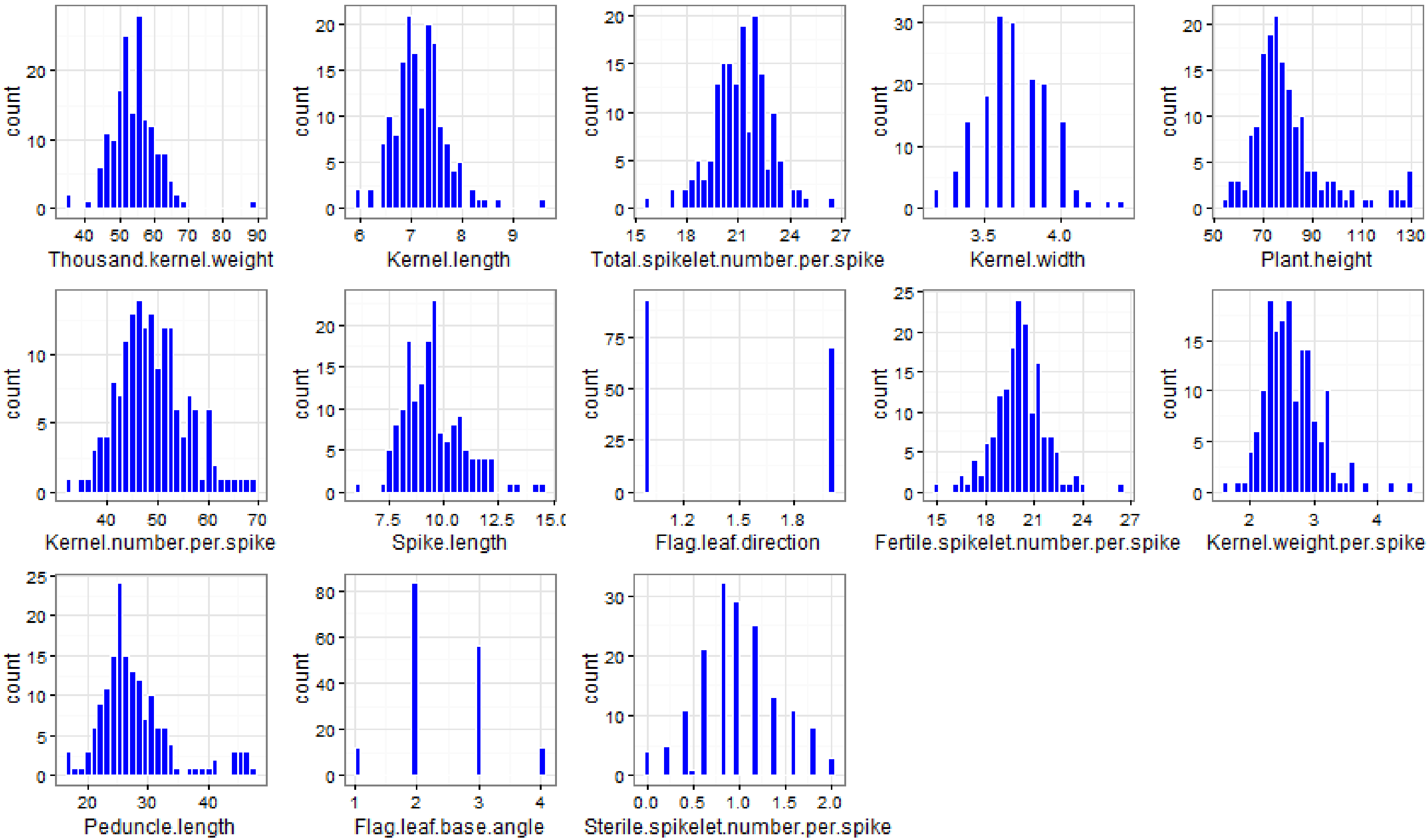

Fig. S1-9 Frequency distribution of phenotypic variation of investigated agronomic traits in 2014, Zhengzhou, environment 9.

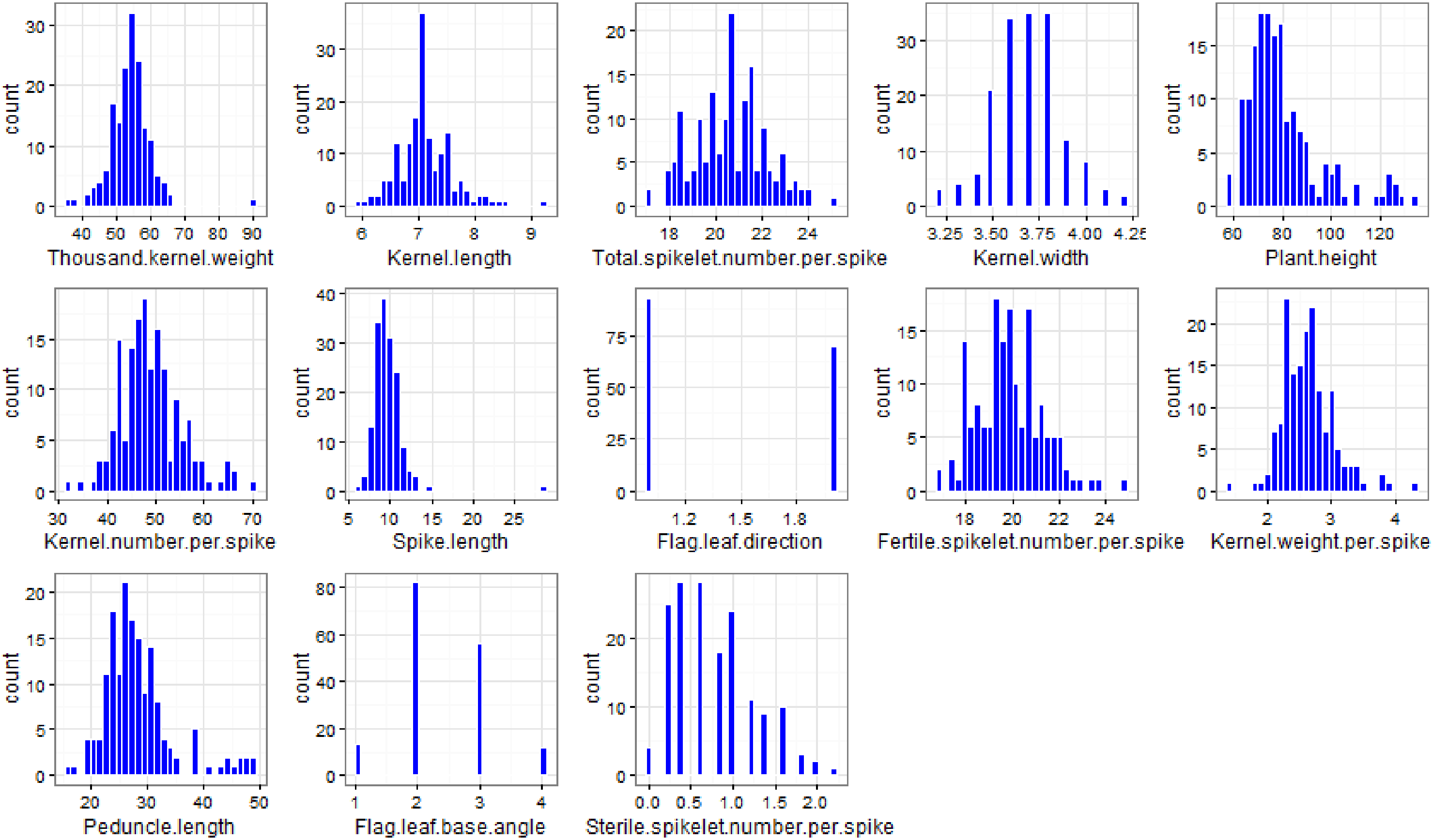

Fig. S1-10 Frequency distribution of phenotypic variation of investigated agronomic traits in 2014, Zhengzhou, environment 10.

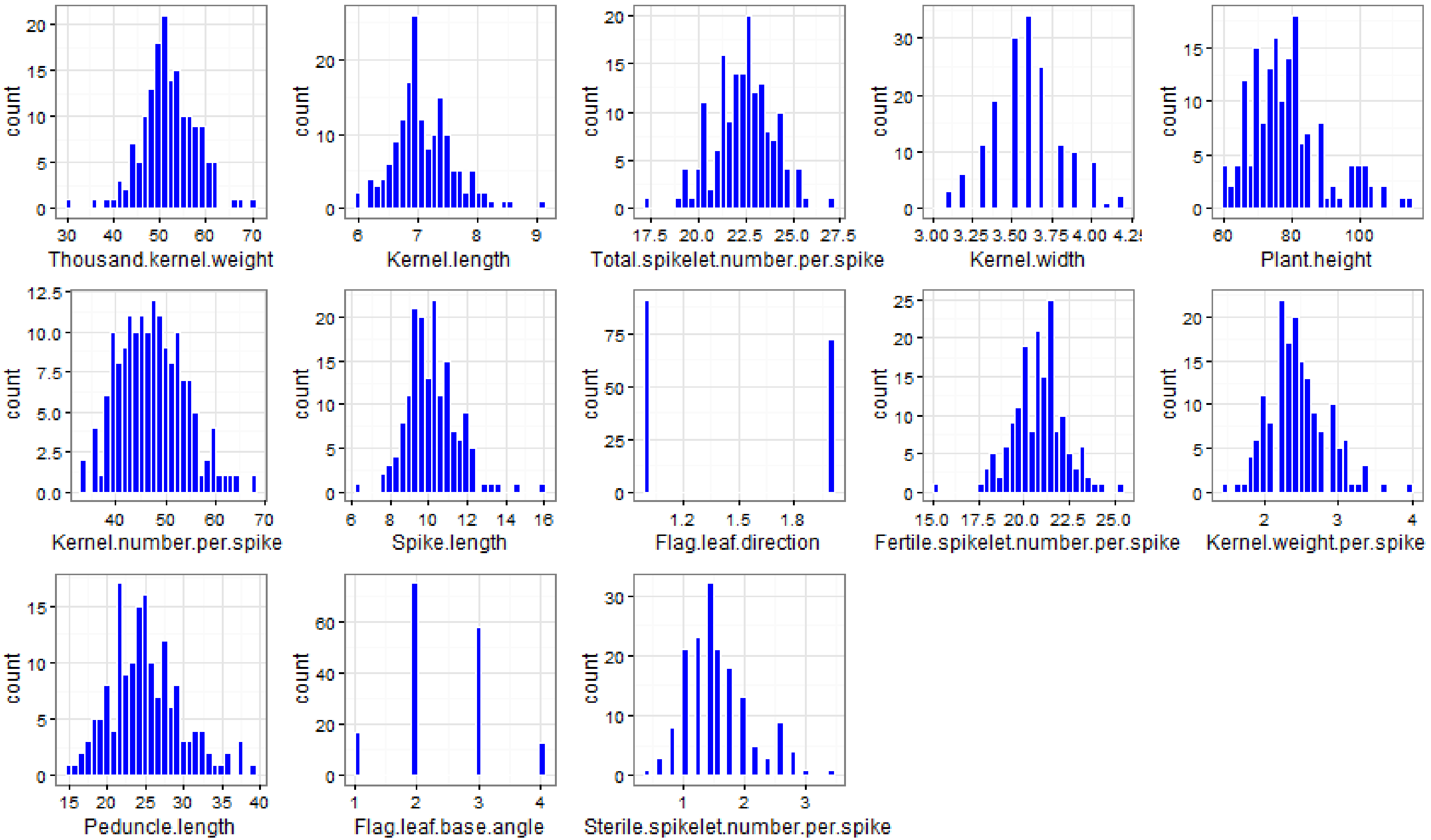

Fig. S1-11 Frequency distribution of phenotypic variation of investigated agronomic traits in 2014, Zhumadian, environment 11.

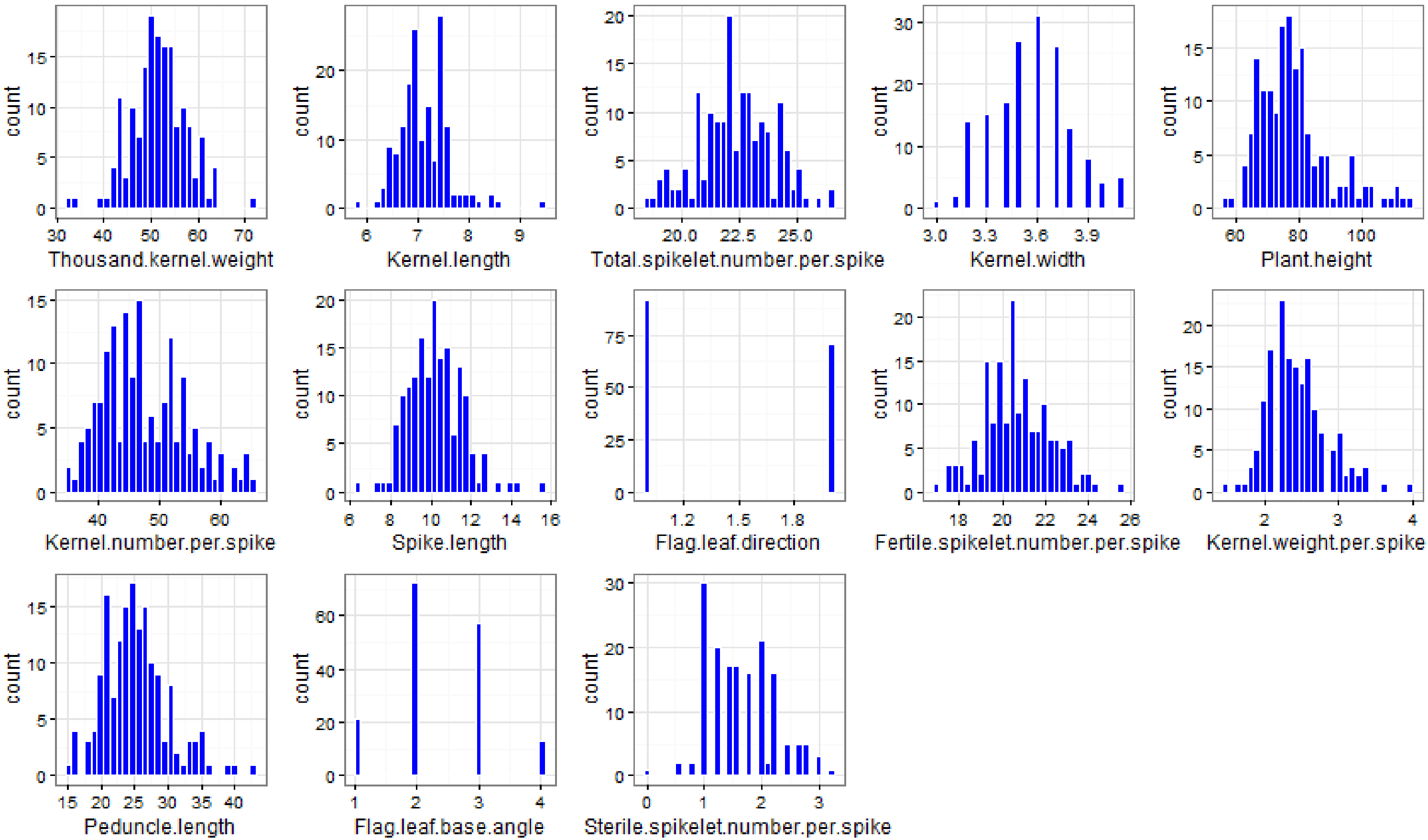

Fig. S1-12 Frequency distribution of phenotypic variation of investigated agronomic traits in 2014, Zhumadian, environment 12.

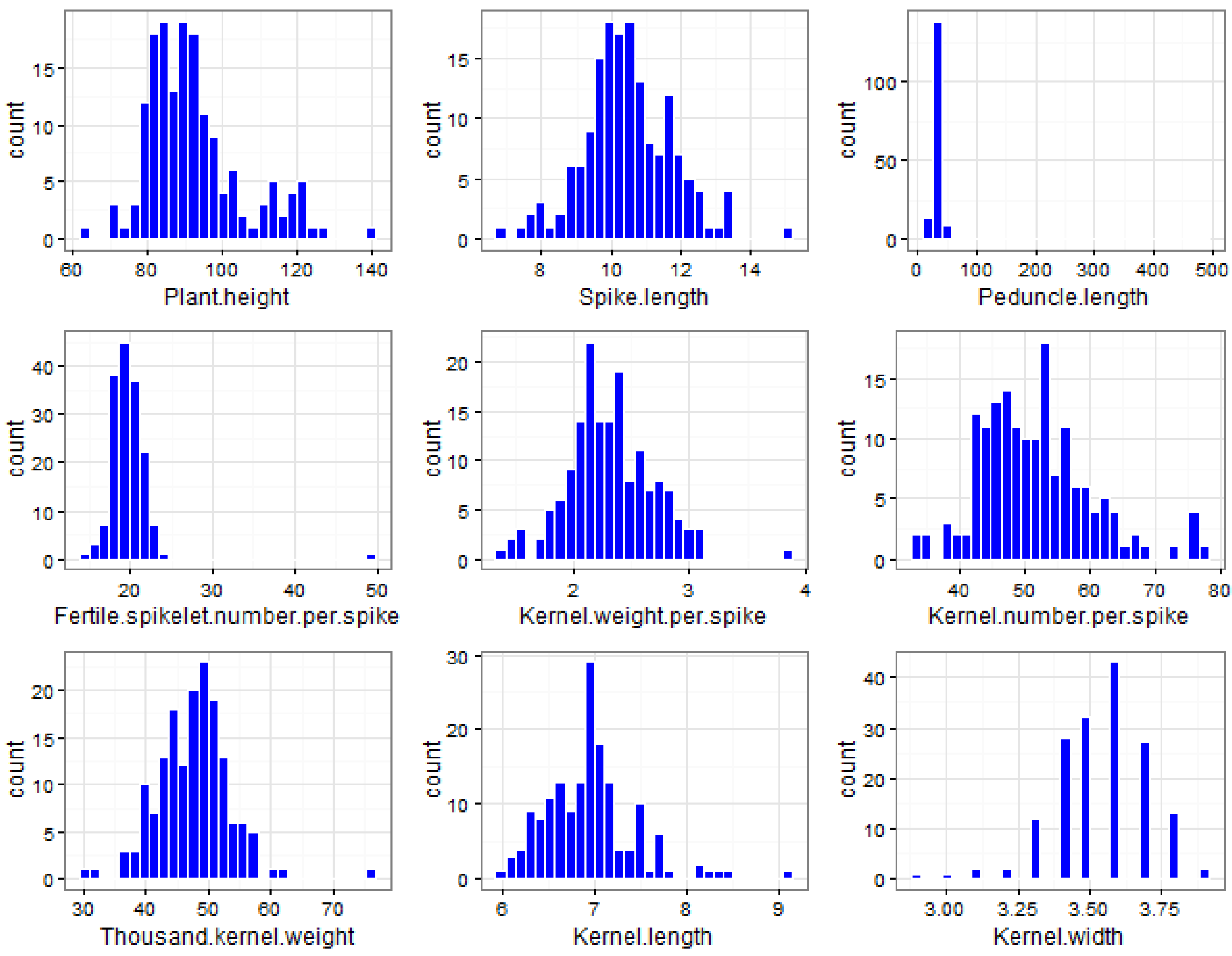

Fig. S1-13 Frequency distribution of phenotypic variation of investigated agronomic traits in 2015, Zhengzhou, environment 13.

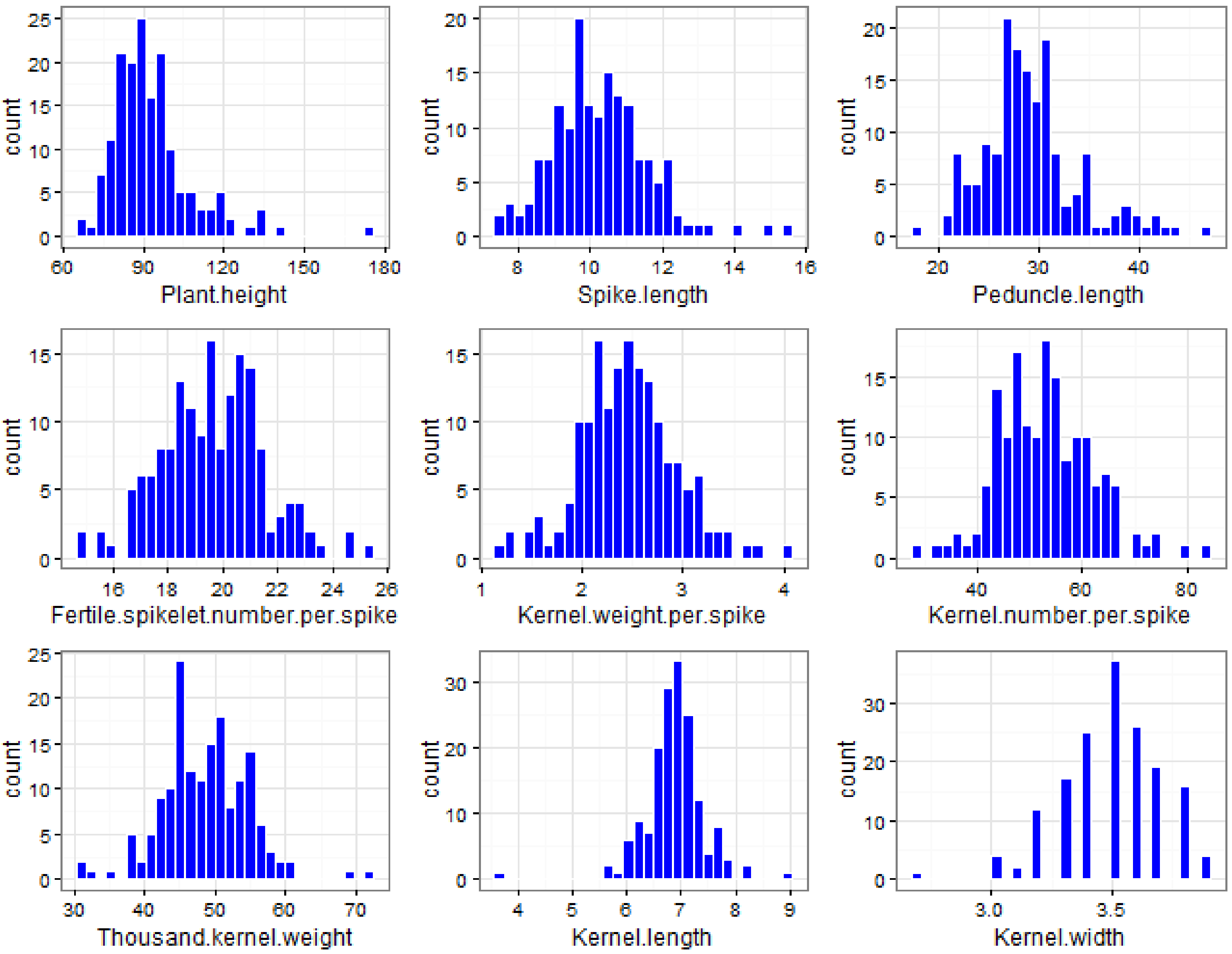

Fig. S1-14 Frequency distribution of phenotypic variation of investigated agronomic traits in 2015, Zhengzhou, environment 14.
