# Supplementary material for: Genome‐wide association study for 13 agronomic traits reveals distribution of superior alleles in bread wheat from the Yellow and Huai Valley of China
Source: Plant Biotechnol J. 2017 Mar 2;15(8):953–69. doi: 10.1111/pbi.12690 (PMC5506658; doi:10.1111/pbi.12690)
Supplement: Supplementary file 2 — Figure S2 Manhattan and quantile–quantile (Q‐Q) plots for 13 agronomic traits. [file PBI-15-953-s007.pdf]

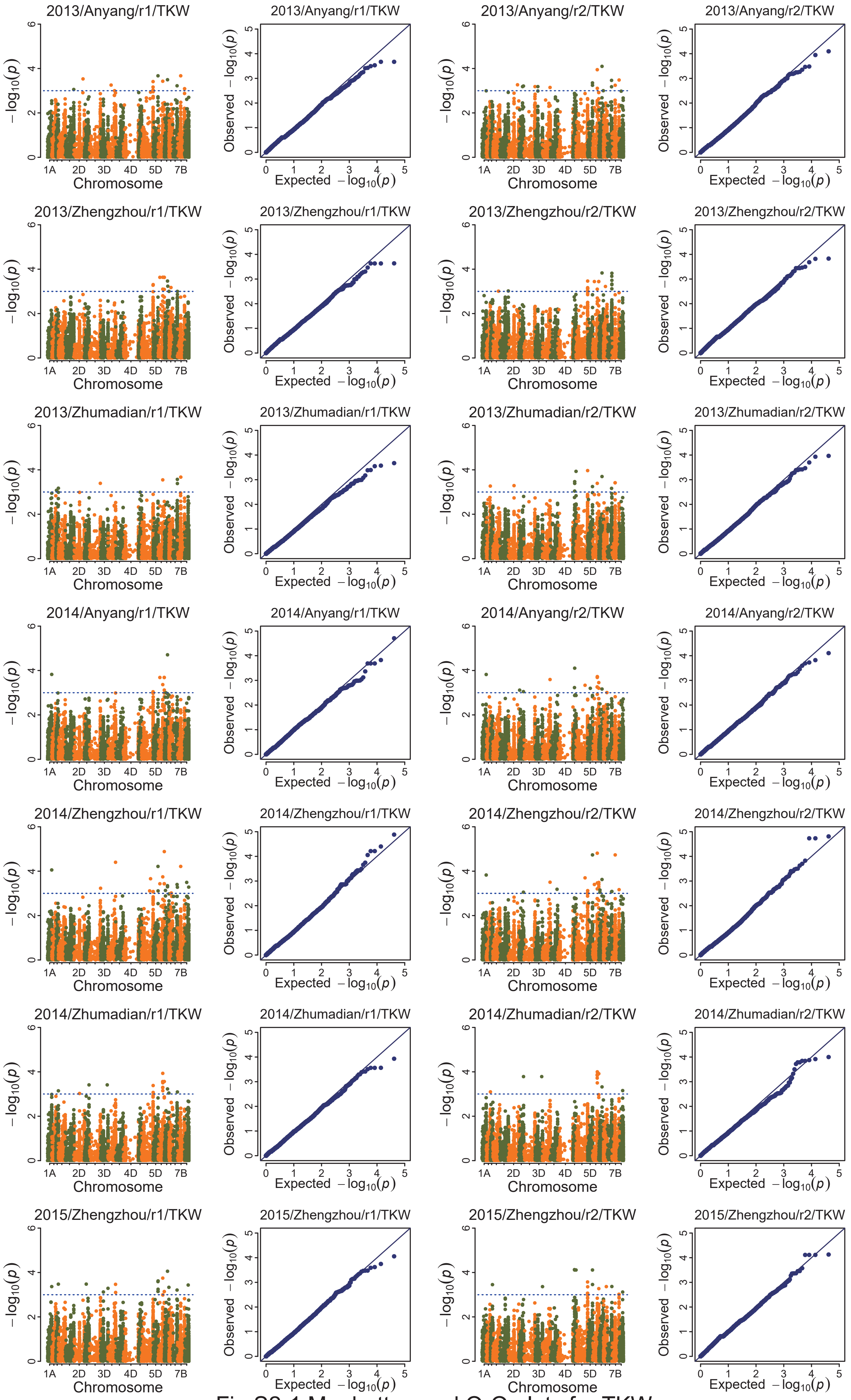

Fig.S2-1 Manhattan and Q-Q plots for TKW.

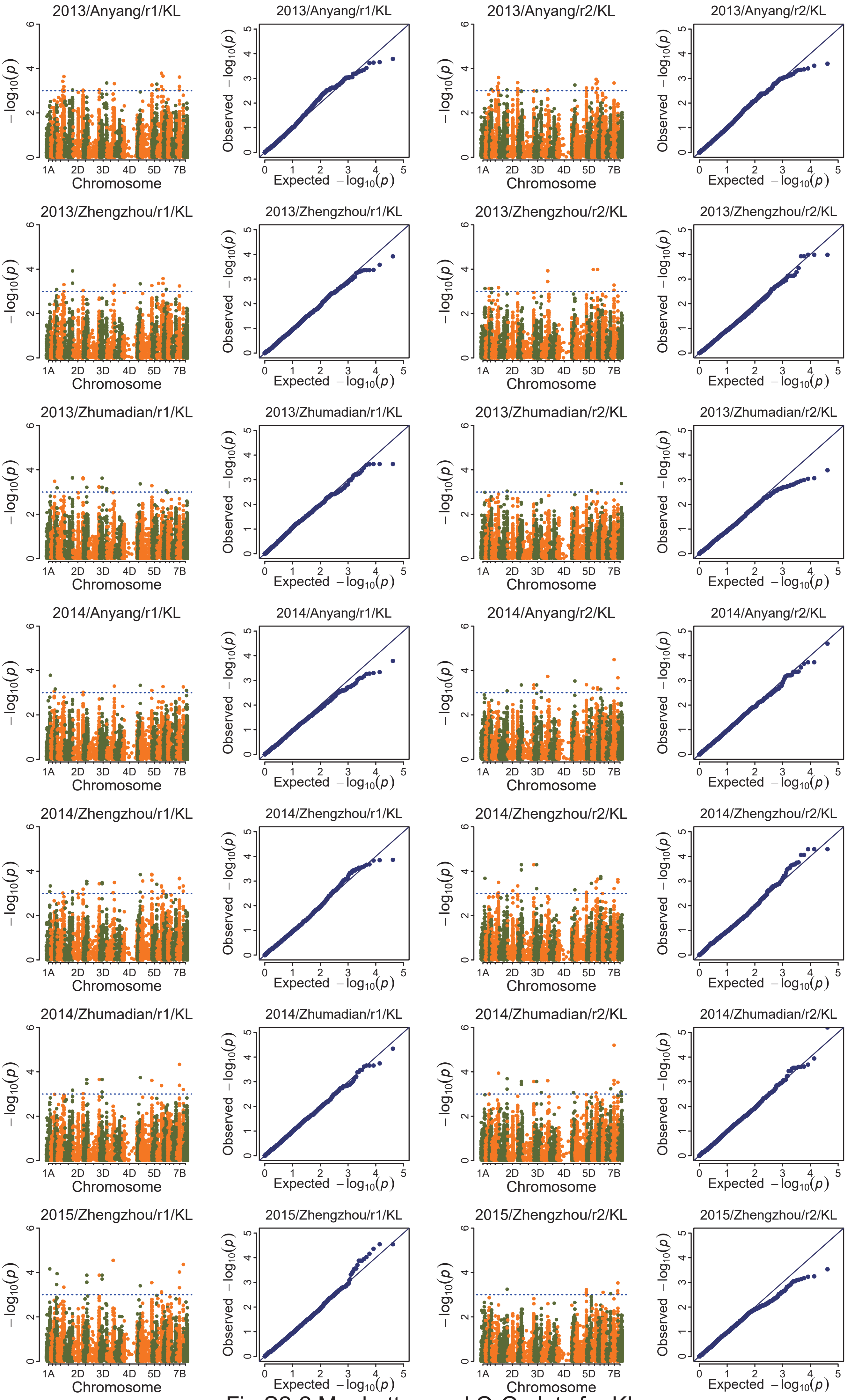

Fig.S2-2 Manhattan and Q-Q plots for KL.

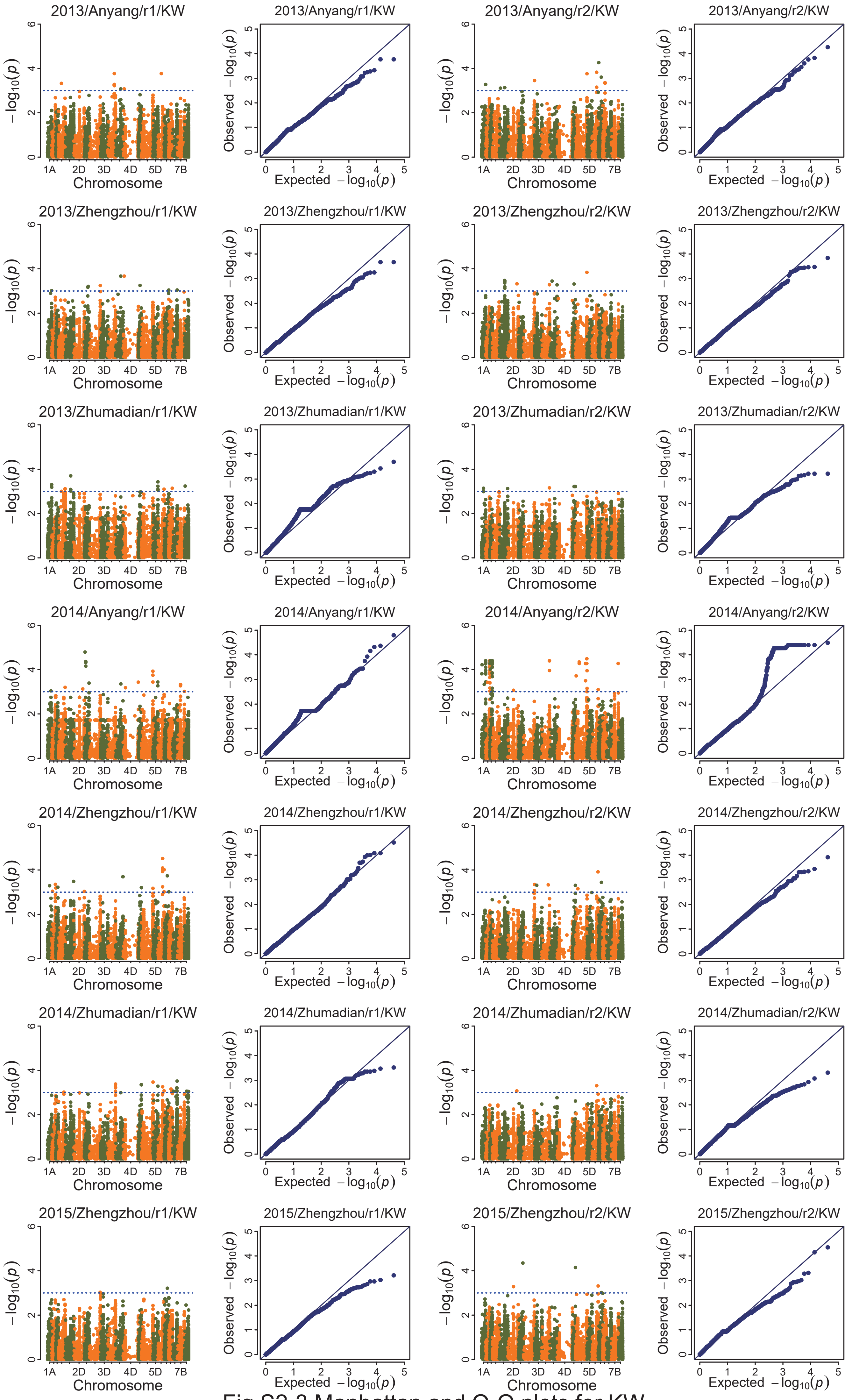

Fig.S2-3 Manhattan and Q-Q plots for KW.

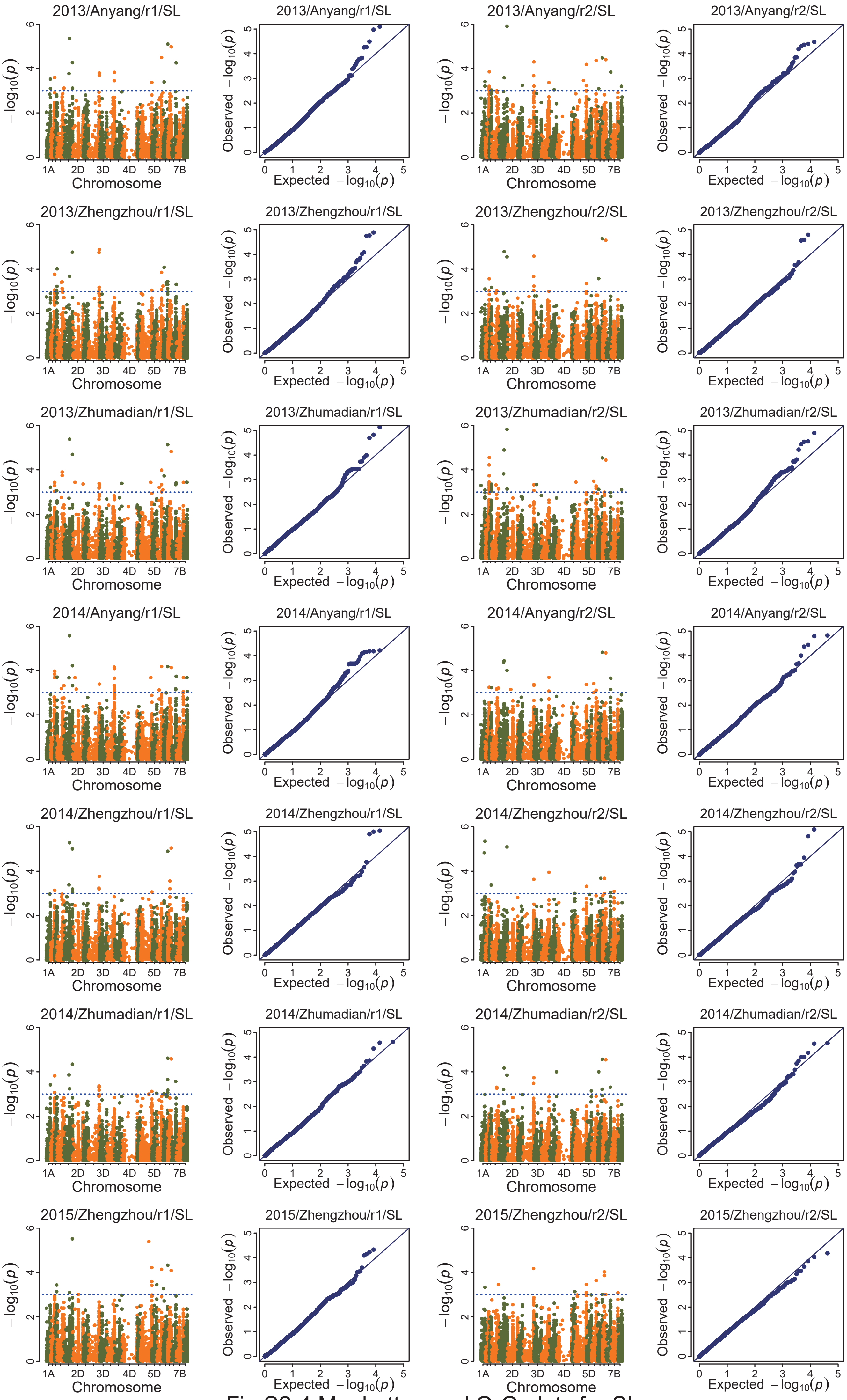

Fig.S2-4 Manhattan and Q-Q plots for SL.

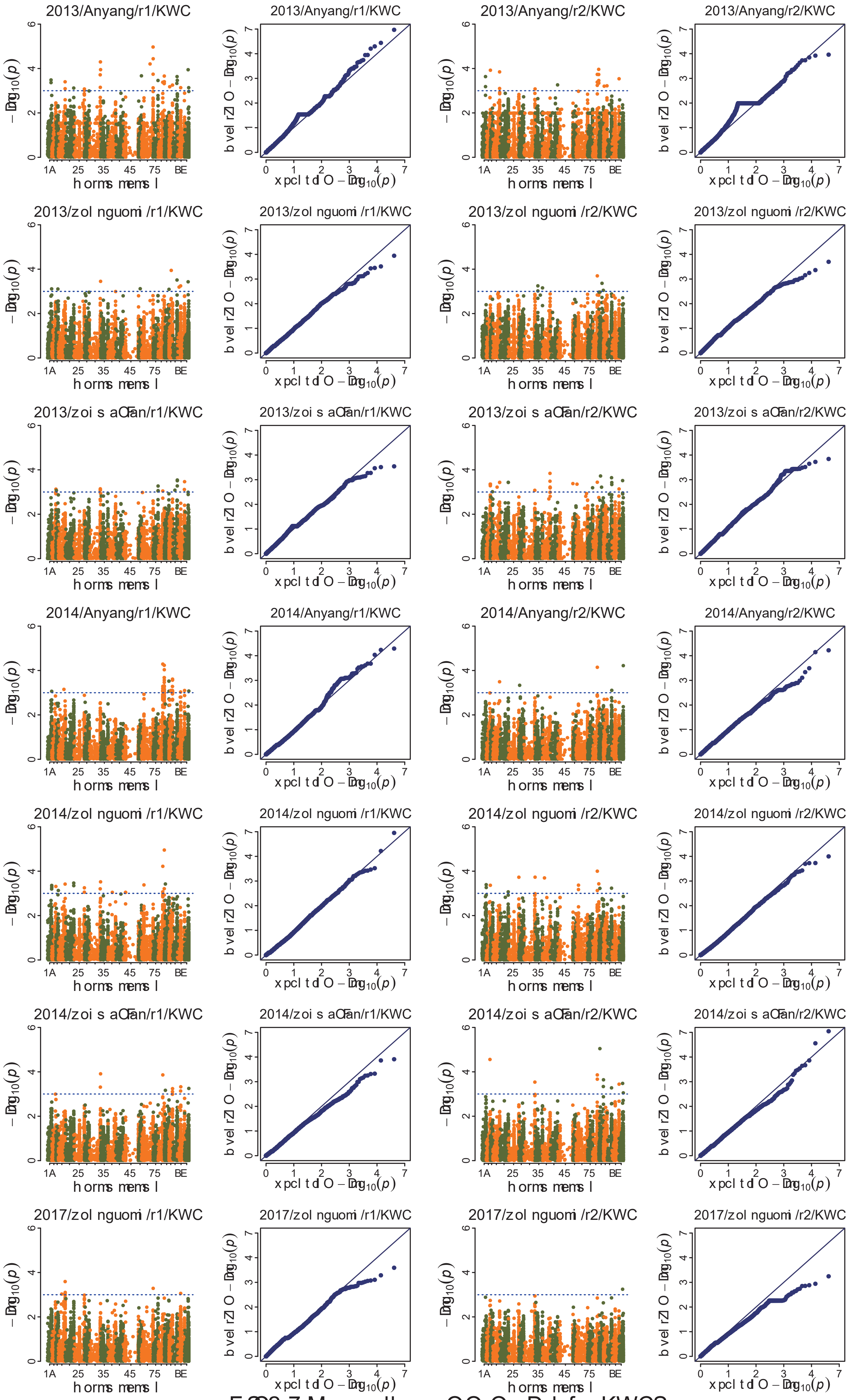

. FgSC2-7 Manoadan anOQ-Q cDnd fmr KWCS

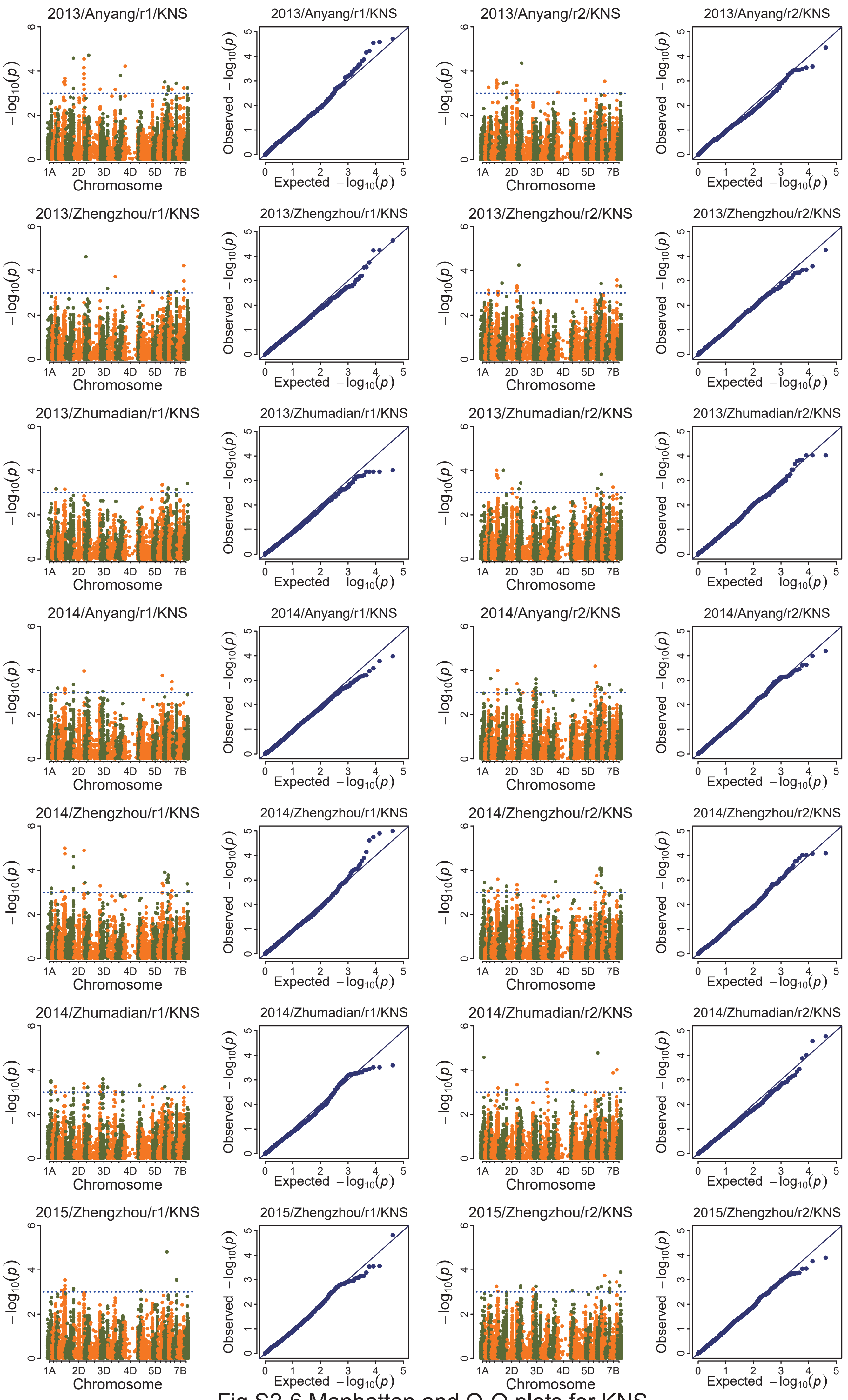

Fig.S2-6 Manhattan and Q-Q plots for KNS.

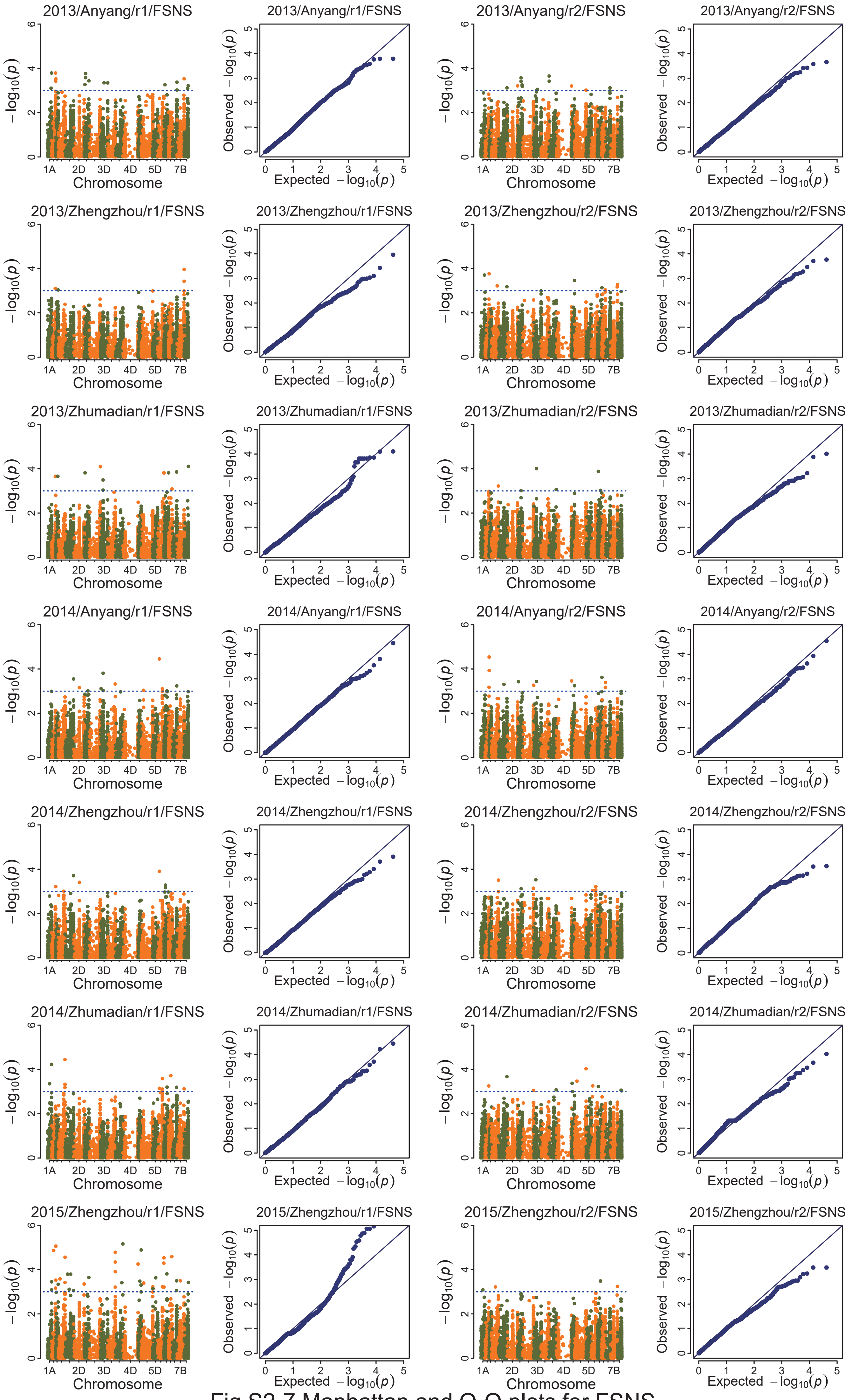

Fig.S2-7 Manhattan and Q-Q plots for FSNS.

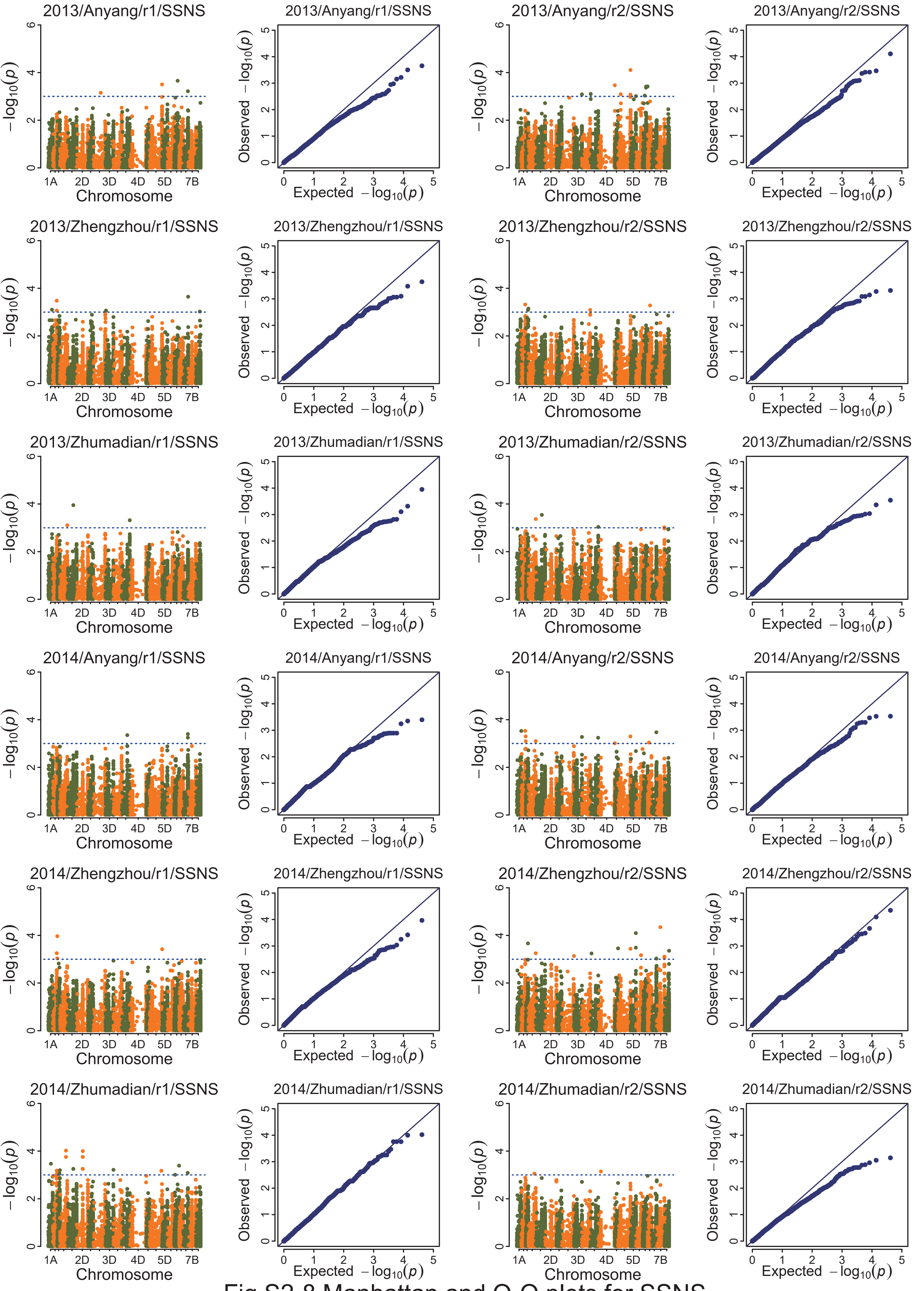

Fig.S2-8 Manhattan and Q-Q plots for SSNS.

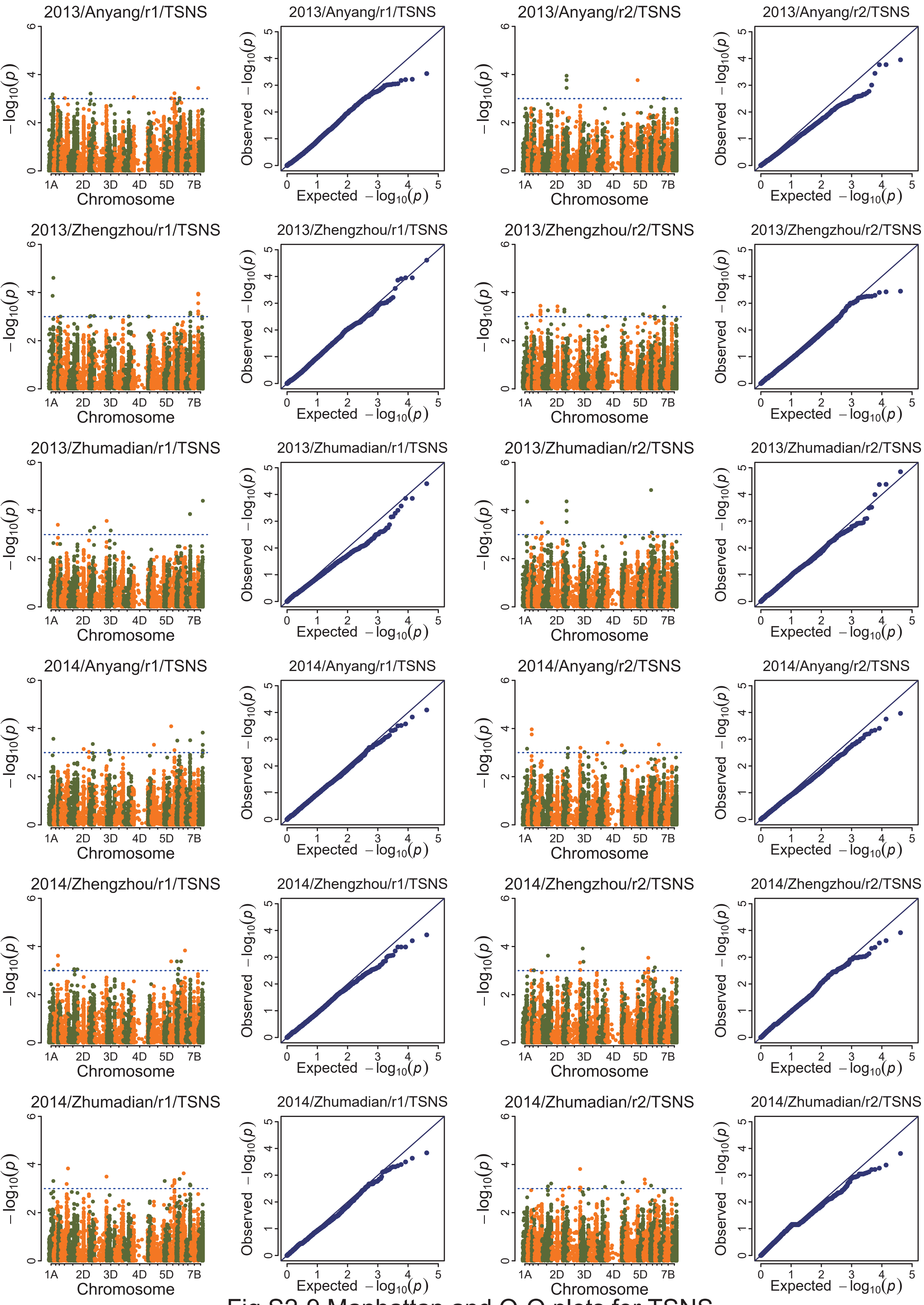

Fig.S2-9 Manhattan and Q-Q plots for TSNS.

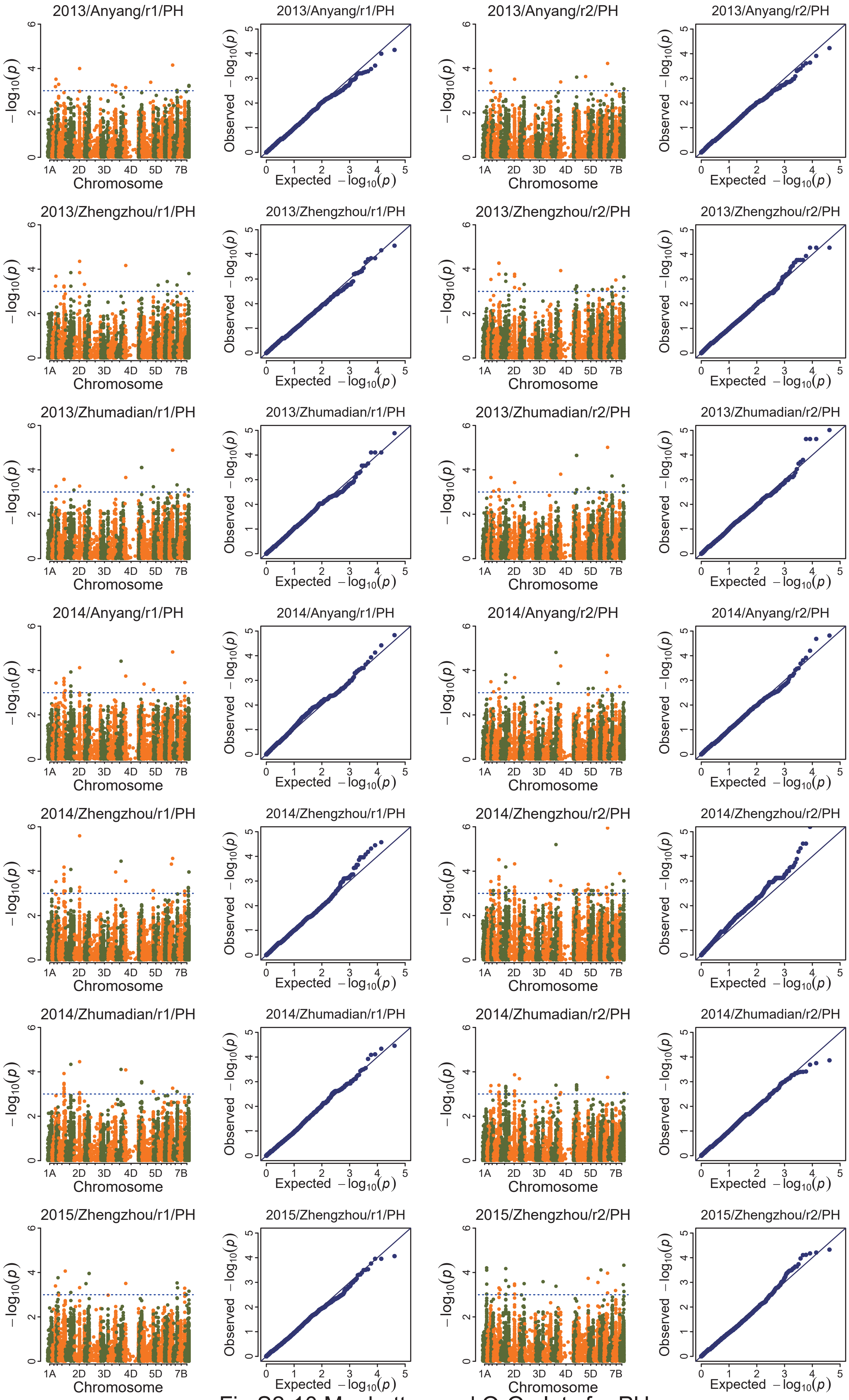

Fig.S2-10 Manhattan and Q-Q plots for PH.

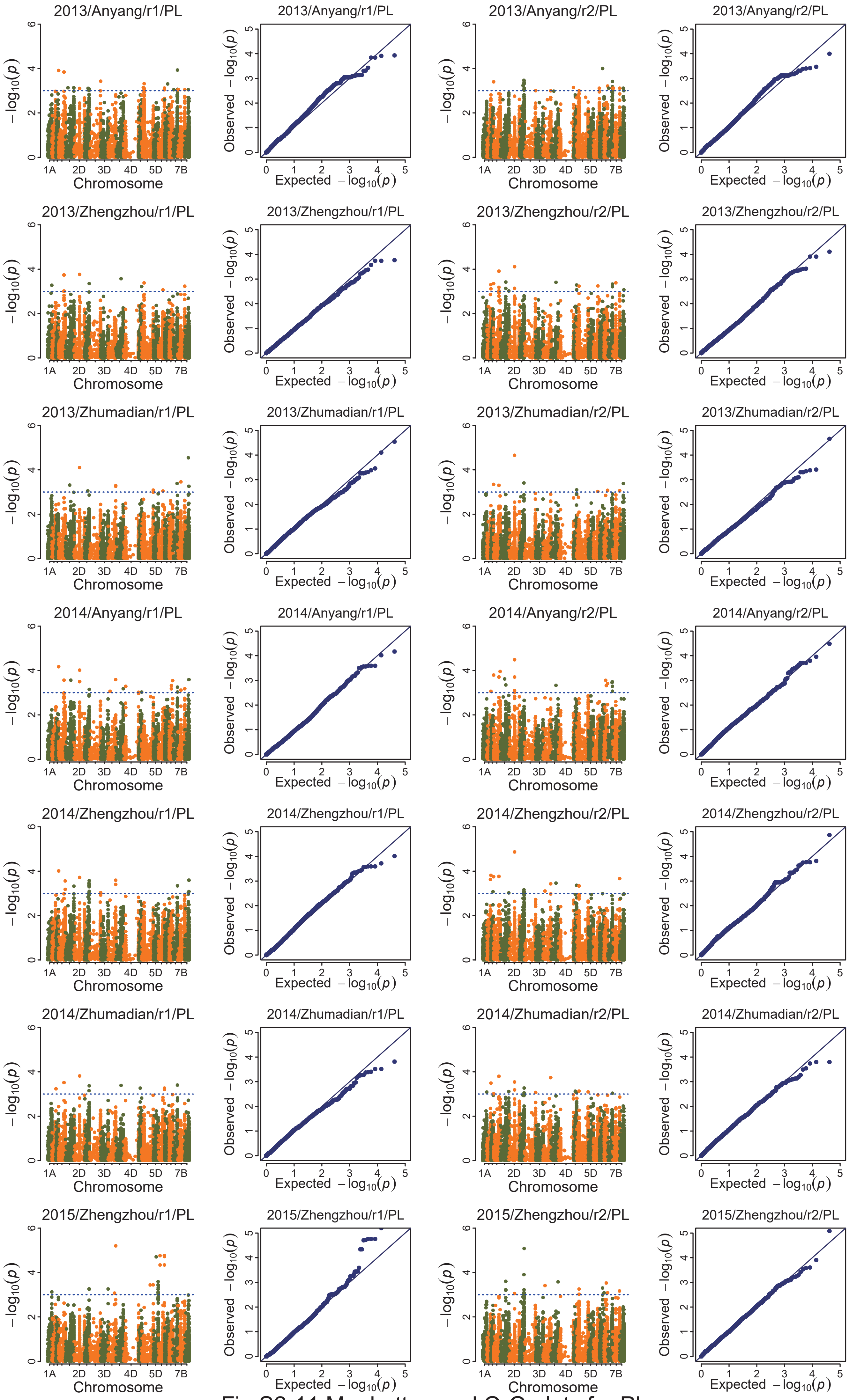

Fig.S2-11 Manhattan and Q-Q plots for PL.

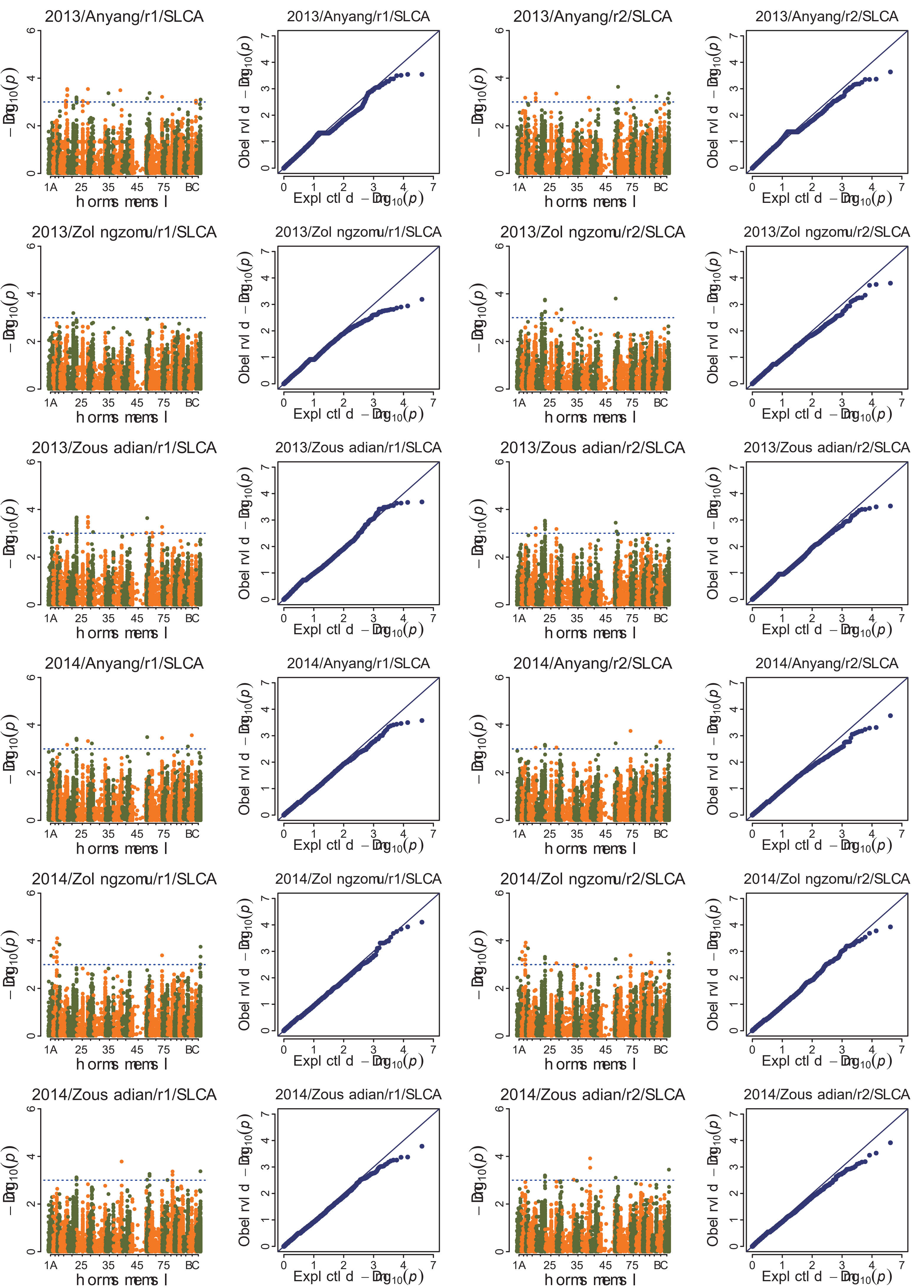

SigF. 2-12 Manoattan and Q-Q pDnte fmr SLCAF

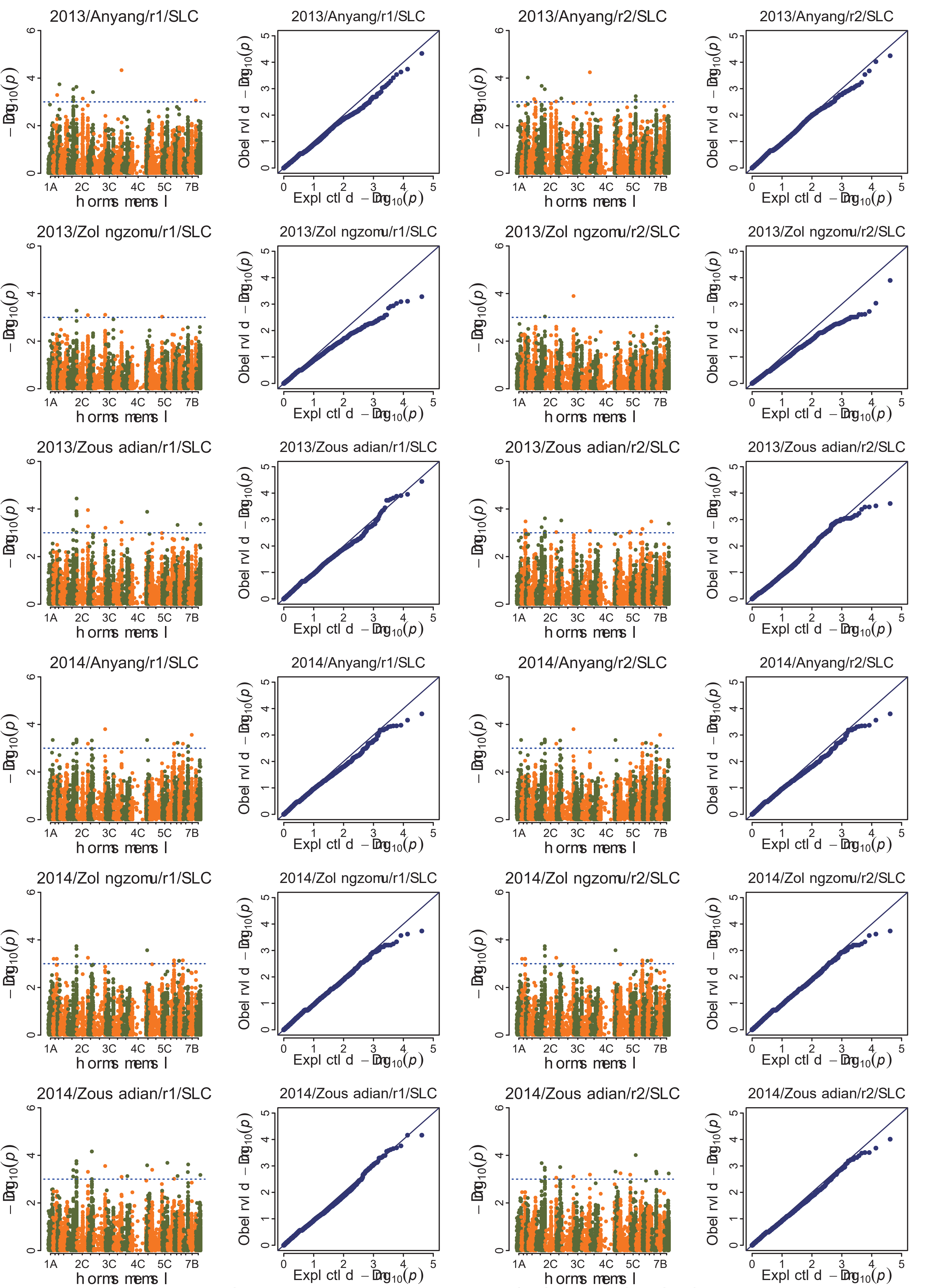

SigF. 2-13 Manoattan and Q-Q pDrte fmr SLCF
