# Supplementary material for: Genome‐wide association study for 13 agronomic traits reveals distribution of superior alleles in bread wheat from the Yellow and Huai Valley of China
Source: Plant Biotechnol J. 2017 Mar 2;15(8):953–69. doi: 10.1111/pbi.12690 (PMC5506658; doi:10.1111/pbi.12690)
Supplement: Supplementary file 3 — Figure S3 Genetic linkage maps of the two RIL populations. (a‐c) Linkage maps for chromosome 3A, 6A and 6B in the PC population; (d) linkage map for chromosome 5B in the UP population. [file PBI-15-953-s006.pdf]

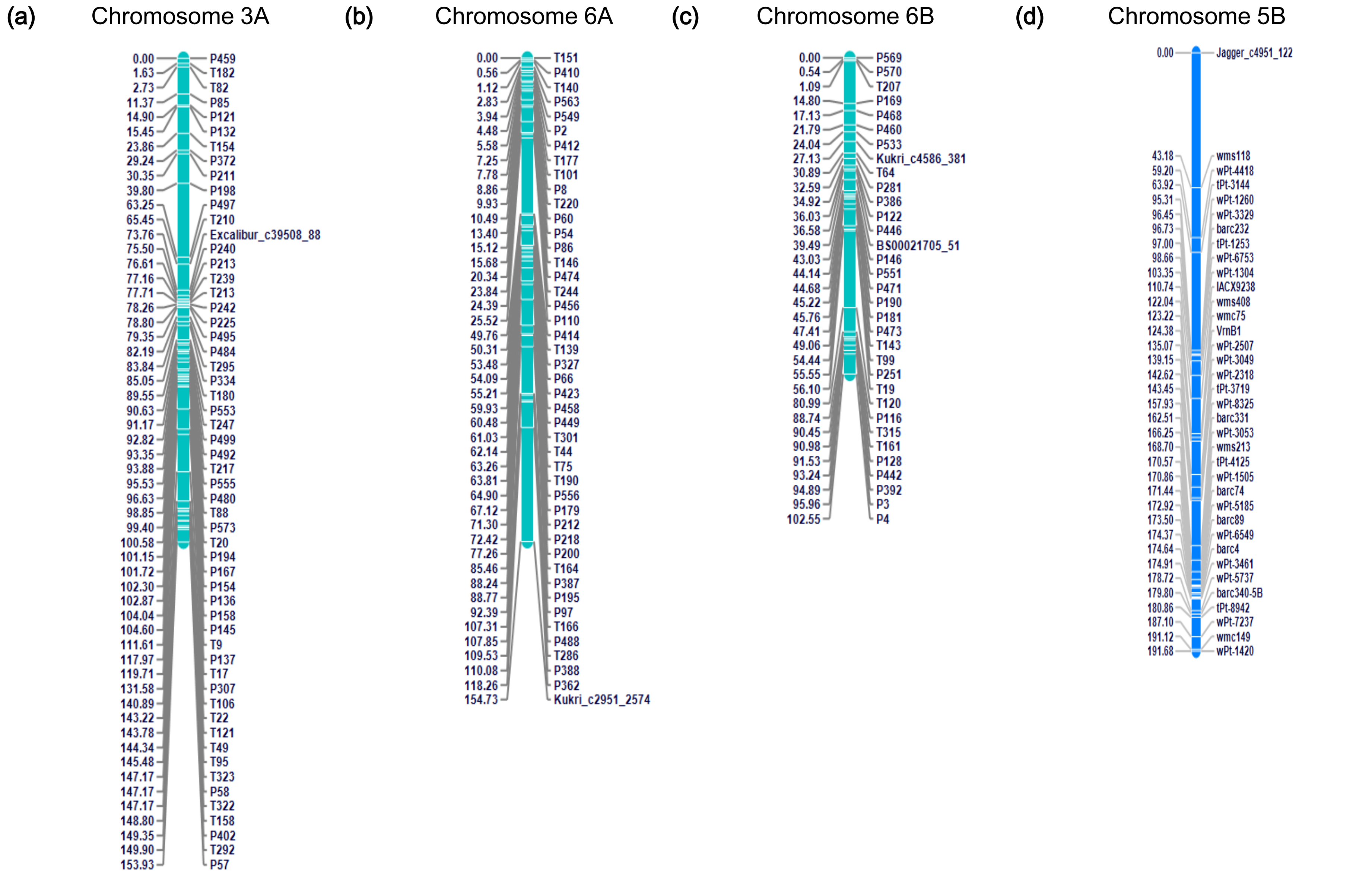

Fig. S3 Genetic linkage maps in the two RIL populations. (a-c) Linkage maps for chromosome 3A, 6A, and 6B in the PC population; (d) Linkage map for chromosome 5B in the UP population.
