# Supplementary figures and images for: Genome‐wide association study for 13 agronomic traits reveals distribution of superior alleles in bread wheat from the Yellow and Huai Valley of China
Source: Plant Biotechnol J. 2017 Mar 2;15(8):953–69. doi: 10.1111/pbi.12690 (PMC5506658; doi:10.1111/pbi.12690)

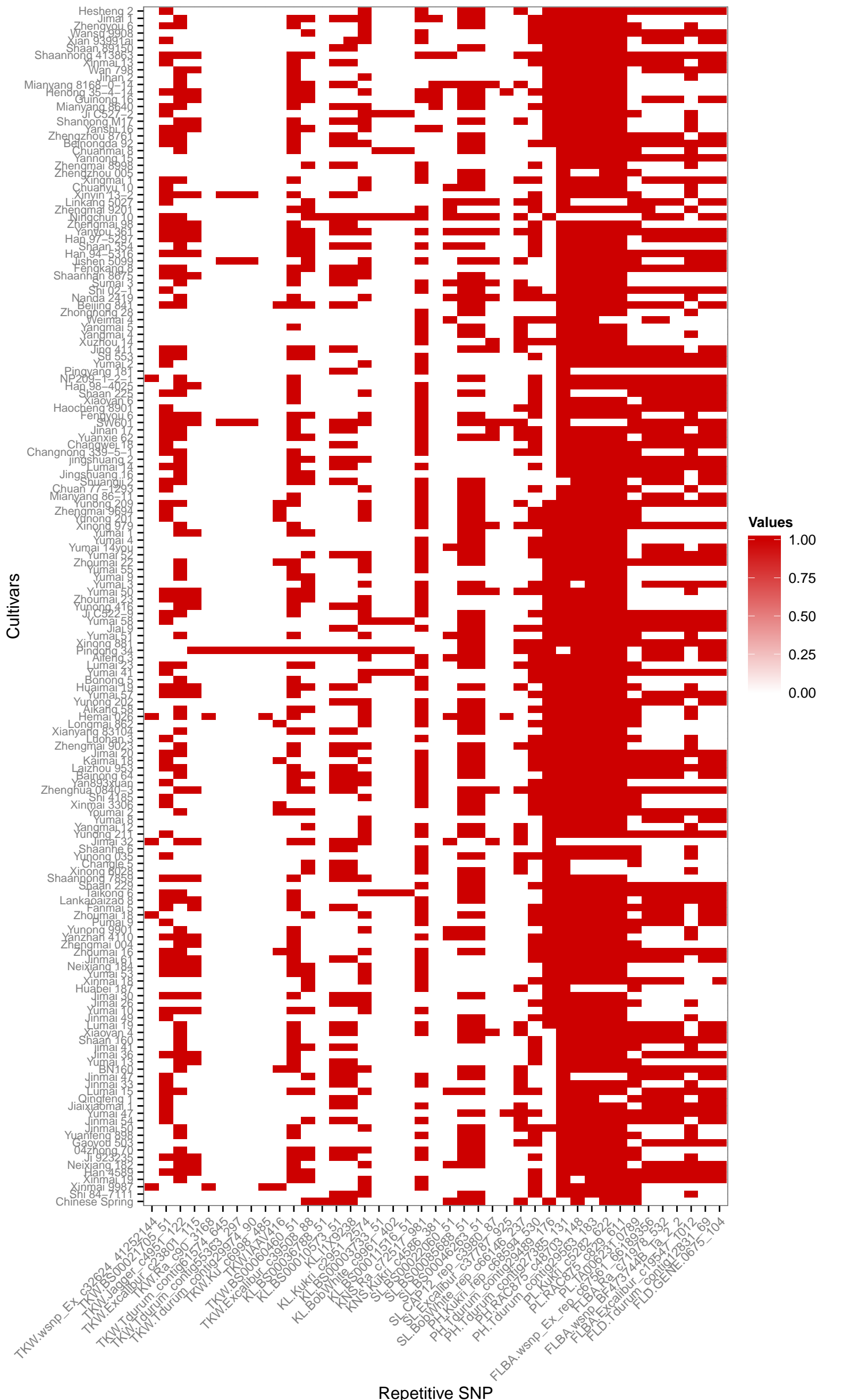

Supplement: Supplementary file 4 — Figure S4 The superior allele loci distributions in the surveyed natural population. Red colours represent superior allele. [file PBI-15-953-s008.pdf]
