# Supplementary material for: Genome‐wide association study for 13 agronomic traits reveals distribution of superior alleles in bread wheat from the Yellow and Huai Valley of China
Source: Plant Biotechnol J. 2017 Mar 2;15(8):953–69. doi: 10.1111/pbi.12690 (PMC5506658; doi:10.1111/pbi.12690)
Supplement: Supplementary file 5 — Table S1 Primers used for detecting significant SNP sites in two RIL populations. [file PBI-15-953-s001.docx]

Table S1 Primers used for detecting significant SNP sites in two RIL populations.

| SNP name | Forward primer | Reverse primer | Expected PCR size | Tm |
| --- | --- | --- | --- | --- |
| Kukri_c2951_2574 | GTAAATACCTTATCCCGATC | GTTTCACTGGATCTAACTC | 528 bp | 52°C |
| Excalibur_c39508_88 | GACTCCCAGGTGCAATAG | CATCTCTCTTTACAATCCC | 410 bp | 52°C |
| BS00021705_51 | GTTTACCAGGCAATCTAATC | CTTTCCTAATACCGCTTAG | 468 bp | 53°C |
| Jagger_c4951_122 | GGAGGTAGACGATATGAG | GATCTCATGAATCCACAAAG | 365 bp | 52°C |

Tm, PCR annealing temperature
