# Supplementary material for: Genome‐wide association study for 13 agronomic traits reveals distribution of superior alleles in bread wheat from the Yellow and Huai Valley of China
Source: Plant Biotechnol J. 2017 Mar 2;15(8):953–69. doi: 10.1111/pbi.12690 (PMC5506658; doi:10.1111/pbi.12690)
Supplement: Supplementary file 7 — Table S3 Significant SNP numbers in pairwise traits. [file PBI-15-953-s003.docx]

Table S3 Significant SNP numbers in pairwise traits.

|  | FLD | FLBA | KL | KNS | KWS | KW | PH | SL | FSNS | SSNS | TKW | PL | TSNS |
| --- | --- | --- | --- | --- | --- | --- | --- | --- | --- | --- | --- | --- | --- |
| Flag leaf direction (FLD) | 89 | 23 | 2 | 0 | 8 | 0 | 0 | 2 | 0 | 0 | 0 | 2 | 0 |
| Flag leaf base angle (FLBA) |  | 86 | 1 | 0 | 0 | 4 | 0 | 0 | 2 | 1 | 0 | 1 | 4 |
| Kernel length (KL) |  |  | 112 | 0 | 4 | 4 | 0 | 0 | 1 | 0 | 17 | 5 | 0 |
| Kernel number per spike (KNS) |  |  |  | 161 | 17 | 1 | 2 | 11 | 14 | 1 | 3 | 1 | 7 |
| Kernel weight per spike (KWS) |  |  |  |  | 167 | 3 | 0 | 16 | 6 | 0 | 7 | 1 | 3 |
| Kernel width (KW) |  |  |  |  |  | 238 | 3 | 0 | 4 | 1 | 22 | 0 | 2 |
| Plant height (PH) |  |  |  |  |  |  | 143 | 2 | 0 | 0 | 2 | 23 | 0 |
| Spike length (SL) |  |  |  |  |  |  |  | 129 | 3 | 3 | 2 | 3 | 4 |
| Fertile spikelet number per spike (FSNS) | |  |  |  |  |  |  |  | 159 | 3 | 2 | 0 | 48 |
| Sterile spikelet number per spike (SSNS) |  |  |  |  |  |  |  |  |  | 81 | 0 | 0 | 1 |
| Thousand kernel weight (TKW) |  |  |  |  |  |  |  |  |  |  | 121 | 3 | 1 |
| Peduncle length (PL) |  |  |  |  |  |  |  |  |  |  |  | 167 | 0 |
| Total spikelet number per spike (TSNS) |  |  |  |  |  |  |  |  |  |  |  |  | 116 |
