# Supplementary material for: Genome‐wide association study for 13 agronomic traits reveals distribution of superior alleles in bread wheat from the Yellow and Huai Valley of China
Source: Plant Biotechnol J. 2017 Mar 2;15(8):953–69. doi: 10.1111/pbi.12690 (PMC5506658; doi:10.1111/pbi.12690)
Supplement: Supplementary file 8 — Table S4 The distribution of focused SNPs superior alleles in the natural population. [file PBI-15-953-s009.docx]

Table S4 The distribution of focused SNPs superior alleles in the natural population.

|  |  |  | Allele type | | Phenotype value | | Allele number | | Allele percentage (%) | |
| --- | --- | --- | --- | --- | --- | --- | --- | --- | --- | --- |
| Trait | SNPname | Chromosome | Inferior | Superior | Inferior | Superior | Inferior | Superior | Inferior | Superior |
| TKW | wsnp_Ex_c32624_41252144 | 6A | GG | TG | 48.7a | 52.9a | 157 | 5 | 96.91 | 3.09 |
|  | BS00021705_51 | 6B | CC | AC | 47.1B | 50.2A | 75 | 81 | 48.08 | 51.92 |
|  | Jagger_c4951_122 | 5B | TC | TT | 47.5B | 50.2A | 69 | 93 | 42.59 | 57.41 |
|  | Excalibur_c23801_115 | 5B | TT | TC | 48.1B | 51.9A | 124 | 39 | 76.07 | 23.93 |
|  | Ra_c90_3168 | 6A | CC | TT | 48.7a | 62.2a | 157 | 3 | 98.13 | 1.88 |
|  | Tdurum_contig61574_645 | 6A | AA | GG | 48.8a | 56.6a | 157 | 4 | 97.52 | 2.48 |
|  | Tdurum_contig55363_297 | 6A | TT | CC | 48.8a | 56.6a | 157 | 4 | 97.52 | 2.48 |
|  | Tdurum_contig29974_90 | 6A | GG | AA | 48.8a | 56.6a | 157 | 4 | 97.52 | 2.48 |
|  | Ku_c6998_485 | 6A | CC | AA | 48.7a | 62.2a | 158 | 3 | 98.14 | 1.86 |
|  | IAAV416 | 7A | CC | GC | 48.5b | 55.0a | 149 | 13 | 91.98 | 8.02 |
|  | BS00060460_51 | 5B | TG | GG | 46.9B | 50.3A | 55 | 100 | 35.48 | 64.52 |
|  | Excalibur_c39508_88 | 3A | GG | AG | 48.3b | 50.2a | 101 | 58 | 63.52 | 36.48 |
| KL | BS00036788_51 | 7B | GG | AA | 7.0a | 7.8a | 159 | 3 | 98.15 | 1.85 |
|  | BS00010573_51 | 5A | CC | AC | 6.9B | 7.2A | 100 | 62 | 61.73 | 38.27 |
|  | IACX9238 | 5B | CC | AC | 6.9B | 7.2A | 100 | 63 | 61.35 | 38.65 |
|  | Kukri_c2951_2574 | 6A | AG | GG | 6.9B | 7.1A | 79 | 75 | 51.30 | 48.70 |
|  | BS00003733_51 | 3A | CC | TC | 7.0a | 7.4a | 156 | 7 | 95.71 | 4.29 |
|  | BobWhite_c9961_402 | 3B | CC | TC | 7.0a | 7.4a | 156 | 7 | 95.71 | 4.29 |
|  | BS00011516_51 | 3D | GG | AG | 7.0a | 7.4a | 156 | 7 | 95.71 | 4.29 |
| KNS | Ra_c72517_981 | 2D | AA | AG | 45.9b | 49.0a | 54 | 102 | 34.62 | 65.38 |
|  | Kukri_c4586_381 | 6B | GG | AG | 47.8b | 55.9a | 156 | 7 | 95.71 | 4.29 |
| SL | BS00022060_51 | 2B | CC | TC | 9.7B | 10.9A | 135 | 21 | 86.54 | 13.46 |
|  | BS00085688_51 | 6B | TC | CC | 9.3B | 10.2A | 62 | 100 | 38.27 | 61.73 |
|  | BS00046263_51 | 6D | TC | CC | 9.3B | 10.2A | 58 | 100 | 36.71 | 63.29 |
|  | CAP12_rep_c3980_87 | 2B | TT | TC | 9.7b | 11.0a | 131 | 17 | 88.51 | 11.49 |
|  | Excalibur_c37787_925 | 3B | GG | AA | 9.8b | 12.2a | 158 | 4 | 97.53 | 2.47 |
|  | BobWhite_rep_c66146_237 | 1B | GG | TG | 9.7B | 10.3A | 110 | 51 | 68.32 | 31.68 |
| PH | Kukri_rep_c68594_530 | 4D | AA | AG | 87.4A | 78.3B | 70 | 91 | 43.48 | 56.52 |
|  | Tdurum_contig29489_176 | 6D | TC | TT | 84.0a | 81.3a | 66 | 97 | 40.49 | 59.51 |
|  | Tdurum_contig27385_131 | 1B | AA | GG | 123.0b | 81.5a | 3 | 158 | 1.86 | 98.14 |
|  | RAC875_c48703_148 | 2D | TC | TT | 112.0B | 80.5A | 9 | 150 | 5.66 | 94.34 |
|  | Tdurum_contig29563_183 | 4B | TC | CC | 109.0B | 80.2A | 11 | 151 | 6.79 | 93.21 |
| PL | Kukri_c5282_622 | 2A | TG | GG | 37.0A | 25.8B | 10 | 151 | 6.21 | 93.79 |
|  | RAC875_c829_611 | 2A | AG | GG | 37.0A | 25.8B | 10 | 151 | 6.21 | 93.79 |
|  | TA006231-0789 | 7A | TC | CC | 29.1A | 26B | 64 | 97 | 39.75 | 60.25 |
| FLBA | wsnp_Ex_rep_c67561_66189356 | 2B | TT | TC | 2.7A | 2.2B | 78 | 85 | 47.85 | 52.15 |
|  | Ra_c71978_532 | 5A | CC | TC | 2.7A | 2.2B | 78 | 84 | 48.15 | 51.85 |
|  | wsnp_BF473744B_Ta_2_2 | 2B | TG | GG | 2.7A | 2.2B | 78 | 83 | 48.45 | 51.55 |
|  | Excalibur_c19547_1012 | 4D | AG | GG | 2.6a | 2.3a | 67 | 92 | 42.14 | 57.86 |
| FLD | Tdurum_contig12831_69 | 2D | AC | CC | 1.7A | 1.4B | 79 | 83 | 48.77 | 51.23 |
|  | GENE-0675_104 | 2B | TC | TT | 1.7A | 1.4B | 79 | 84 | 48.47 | 51.53 |
