# Supplementary material for: Genome‐wide association study for 13 agronomic traits reveals distribution of superior alleles in bread wheat from the Yellow and Huai Valley of China
Source: Plant Biotechnol J. 2017 Mar 2;15(8):953–69. doi: 10.1111/pbi.12690 (PMC5506658; doi:10.1111/pbi.12690)
Supplement: Supplementary file 9 — Table S5 P value of t‐test for averaged TKW grouped by taking different number of superior alleles. [file PBI-15-953-s005.docx]

Table S5 P value of T-test for averaged TKW grouped by taking different number of superior alleles.

|  |  |  |  |  | 2013 | | | | | | | | | 2014 | | | | | | | | | 2015 | | |
| --- | --- | --- | --- | --- | --- | --- | --- | --- | --- | --- | --- | --- | --- | --- | --- | --- | --- | --- | --- | --- | --- | --- | --- | --- | --- |
|  |  |  |  |  | Anyang | | | Zhengzhou | | | Zhumadian | | | Anyang | | | Zhengzhou | | | Zhumadian | | | Zhengzhou | | |
| SNP1 | SNP2 | SNP3 | Number | | Phenotype | | P | Phenotype | | P | Phenotype | | P | Phenotype | | P | Phenotype | | P | Phenotype | | P | Phenotype | | P |
| AC | TC | GG | 26 | 20 | 46.61 | 43.54 | 0.05 | 42.52 | 38.52 | 0.01* | 41.57 | 38.95 | 0.10 | 53.67 | 49.15 | 0.00** | 53.93 | 48.85 | 0.00** | 51.15 | 46.41 | 0.00** | 48.00 | 43.87 | 0.00** |
| CC | TT | GG | 24 | 20 | 46.55 | 43.54 | 0.06 | 43.16 | 38.52 | 0.00** | 42.08 | 38.95 | 0.07 | 52.57 | 49.15 | 0.03* | 53.10 | 48.85 | 0.01* | 50.46 | 46.41 | 0.02* | 46.93 | 43.87 | 0.03* |
| CC | TC | AG | 13 | 20 | 45.87 | 43.54 | 0.25 | 41.65 | 38.52 | 0.11 | 43.37 | 38.95 | 0.06 | 53.81 | 49.15 | 0.02* | 51.43 | 48.85 | 0.24 | 50.32 | 46.41 | 0.14 | 47.83 | 43.87 | 0.03* |
| AC | TT | GG | 26 | 20 | 50.43 | 43.54 | 0.00** | 46.53 | 38.52 | 0.00** | 46.15 | 38.95 | 0.00** | 56.39 | 49.15 | 0.00** | 55.82 | 48.85 | 0.00** | 54.06 | 46.41 | 0.00** | 49.45 | 43.87 | 0.00** |
| AC | TC | AG | 3 | 20 | 50.27 | 43.54 | 0.11 | 40.47 | 38.52 | 0.63 | 42.53 | 38.95 | 0.45 | 53.24 | 49.15 | 0.00** | 54.02 | 48.85 | 0.01* | 51.27 | 46.41 | 0.00** | 44.45 | 43.87 | 0.66 |
| CC | TT | AG | 16 | 20 | 48.35 | 43.54 | 0.00** | 43.95 | 38.52 | 0.00** | 42.59 | 38.95 | 0.05 | 55.08 | 49.15 | 0.00** | 53.63 | 48.85 | 0.01* | 52.30 | 46.41 | 0.00** | 47.71 | 43.87 | 0.02* |
| AC | TT | AG | 24 | 20 | 50.86 | 43.54 | 0.00** | 45.46 | 38.52 | 0.00** | 45.88 | 38.95 | 0.00** | 57.00 | 49.15 | 0.00** | 56.15 | 48.85 | 0.00** | 53.85 | 46.41 | 0.00** | 50.39 | 43.87 | 0.00** |

SNP1 = BS00021705_51;

SNP2 = Jagger_c4951_122;

SNP3 = Excalibur_c39508_88;

Superior alleles for SNPs BS00021705_51, Jagger_c4951_122, and Excalibur_c39508_88 are AC, TT, and AG, respectively;

P, p value of T-test;

*, P < 0.05; **, P < 0.01; ns, not significant;
